# Epigallocatechin Gallate Enzymatic Alpha Glucosylation Potentiates Its Skin-Lightening Activity—Involvement of Skin Microbiota

**DOI:** 10.3390/molecules29225391

**Published:** 2024-11-15

**Authors:** Cloé Boira, Emilie Chapuis, Laura Lapierre, Daniel Auriol, Cyrille Jarrin, Patrick Robe, Jean Tiguemounine, Amandine Scandolera, Romain Reynaud

**Affiliations:** 1 Givaudan Active Beauty, R&D, 51110 Pomacle, France; 2 Givaudan Active Beauty, R&D, 31400 Toulouse, France; 3 Polyclinique Courlancy, Surgery, 51100 Reims, France

**Keywords:** EGCG, pigmentation, glucoside, melanogenesis, photoaging, trigonelline, *Lactobacillus*

## Abstract

(1) Background: Ultraviolet radiation takes part in photoaging and pigmentation disorders on skin. Epigallocatechin gallate (EGCG) is a well-known brightening and photoprotective compound but it faces limitations in terms of stability and solubility. (2) Methods: A more stable and water-soluble glucoside called EGCG-G1 was obtained by enzymatic glucosylation of EGCG. In vitro and ex vivo experiments evaluated EGCG-G1 skin penetration, antioxidant activity, and antimelanogenic properties compared to EGCG. This gene expression study characterized the pathways impacted by EGCG-G1. Four clinical studies covering phototypes I to V, at various ages, and different skin areas, using several tools, were conducted to assess the effect of EGCG-G1 on skin hyperpigmentation and tone. The impact of glucoside on skin microbiota, especially *Lactobacillus* sp., was assessed through in vitro and in vivo investigations. (3) Results: EGCG-G1 better penetrated the epidermis than EGCG due to a possible interaction with GLUT1. EGCG-G1 presented similar antioxidant activity to that of EGCG and decreased melanogenesis through the inhibition of 13 genes, including MITF. The skin *Lactobacillus* population increased with EGCG-G1, which promoted bacterial growth in vitro as prebiotic, and induced the release of a microbial brightening metabolite. Clinical trials demonstrated EGCG-G1 to decrease hyperpigmented spots and increase skin brightness and homogeneity in a large panel of phototypes, outperforming EGCG and vitamin C. (4) Conclusions: Glucosylation of EGCG maintained its photoprotective antioxidant properties and enhanced penetration across the epidermis. EGCG-G1 demonstrated brightening properties on all skin types by down-regulation of melanogenesis pathways and indirectly by skin microbiota stimulation.

## 1. Introduction

Human skin pigmentation depends on the biosynthesis of the pigment melanin that protect teguments from the deleterious effects of ultraviolet radiation (UVR). The regulation of pigmentation is a complex mechanism involving more than 170 genes. Melanin synthesis occurs in melanocytes and is regulated by extrinsic factors (UVR, pollution) as well as intrinsic stimuli (hormones, inflammation). They can be integrated by keratinocytes and fibroblasts as well, and have a paracrine effect on melanogenesis [1,2]. 

Several signaling pathways meet the pivotal transcription factor MITF (Microphtalmia-associated Transcription Factor), controlling genes involved in biogenesis and the function of melanosome, the specific organelle responsible for melanin synthesis and transfer to neighboring keratinocytes [3,4]. Notably, MITF regulates melanogenic enzymes TYR (Tyrosinase) and TYRP1/2 (Tyrosinase-Related Protein 1 and 2). Two cascade signals are considered core for melanogenesis: the Stem Cell Factor (SCF)/Tyrosine Kinase (KIT) and the alpha-Melanocyte Stimulating Hormone (α-MSH)/MelanoCortin 1 Receptor (MC1R) pathways [1,5].

Skin pigmentation has evolved in the human lineage to adapt to environmental conditions, especially UVR levels’ variation at different latitude and seasons, balancing the photoprotective effect of melanin, with the requirement for UV-B involved in the cutaneous vitamin D synthesis [6]. The genetic background of skin color defines skin phenotypes and plays an important role in the variation in the response to sun exposure and related consequences [7,8,9]. The Fitzpatrick scale determines six skin phototypes according to the response to UVR, from I, the palest skins that always burn and never tan, to VI, the darkest ones that never burn. Two main melanin pigments are synthesized on skin at different mixed ratios depending on skin type: eumelanin, prevalent in phototypes V-VI, which have photoprotective properties; and pheomelanin, responsible for phototype I [10]. Solar radiation, and more particularly UVR, as part of the exposome, increases the risk of skin cancer, especially in light-skin-colored people, and promotes premature aging, called photoaging [8,10,11]. UVR triggers oxidative stress, inflammation, cell senescence and increases extracellular matrix degradation, leading to clinical signs of aging (wrinkles, laxity or roughness and pigmentary changes). Darker skins are less susceptible to sun-induced damage due to a thicker epidermis and higher melanin content [10]. However, all phototypes, and especially the IV to VI, present hyperpigmentation disorders as brown spots, dermatosis papulosa nigra, seborrheic keratosis and solar lentigo [9,12]. Uneven distribution of skin pigment is considered a beauty flaw and there is demand for solutions that keep undesirable hyperpigmentation responses low. Hyperpigmentation depends on UVR exposure intensity and is linked to the polymorphism of traits (melanogenic enzymes, melanoma biogenesis, melanosome transfer, etc.) regulating melanogenesis, which constitute targets for the development of such products appropriate for all skin types [9].

In addition, it has emerged that skin microbiota is influenced by chronological aging [13,14]. Recent works revealed the interplay between UVR, skin and microbiota: UVR exposure modulates skin and gut microbiota; skin metabolome, immune function and response to UVR are influenced by the microbiota [15,16,17,18]. A possible link between microbiota and melanogenesis is also emerging. Hyperpigmented skin presents a specific microbial composition and probiotics were shown to down-regulate melanin synthesis [19,20]. These data open perspectives for the protective role of microbiota in photoaging [21].

Green tea derived from *Camellia sinensis* leaves contains polyphenols and a particularly high content of (–)-epigallocatechin-3-gallate (EGCG) [22]. This natural compound has been known for years to prevent photocarcinogenesis [23]. Its skin-protecting effects against photoaging are described in vitro, as EGCG displays antioxidant activity by decreasing UV-induced reactive oxygen species (ROS) content in cells, decreases hyaluronidase activity limiting extracellular matrix degradation, improves barrier function and inhibits melanogenesis by decreasing MITF and TYR protein content [24,25,26]. EGCG was also proved to reduce the visible signs of photoaging at the clinical level [27]. The use of EGCG is restricted by low water solubility and chemical stability in an aqueous solution. Evidence demonstrates that enzymatic glucosylation is an opportunity to address these limitations without affecting the photoprotective properties of the molecule [28,29,30,31]. However, an enzymatic reaction produces a diversity of glucosides and the obtention of a high concentration of a single glucosylated form remains challenging. 

In this work, a monoglucoside of EGCG (EGCG-G1), more precisely, (–)-epigallocatechin-3-gallate-4′-*O*-α-D-glucoside, was synthesized. The effects of EGCG-G1 on melanogenesis were evaluated ex vivo and in four clinical trials, including volunteers with different ancestry (African, Asian, Caucasian, and Indian), covering a large panel of phototypes, which presented hyperpigmentation. The penetration of EGCG-G1 and impact on microbiota were also addressed in this study. 

## 2. Results

### 2.1. Chemical Structure of EGCG-G1

**Figure 1:**
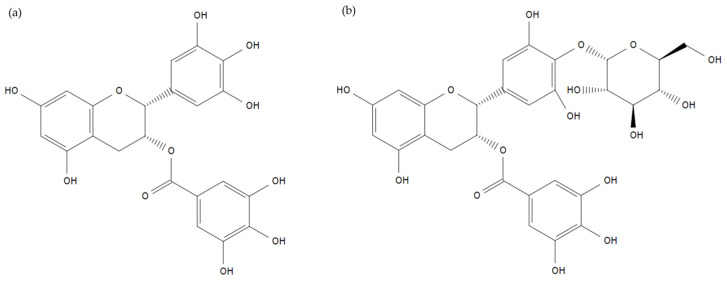
Chemical structure of EGCG (**a**) and (-)-epigallocatechin-3-gallate-4′-O-α-D-glucoside (**b**) making up 70% of EGCG-G1.

NMR analysis of EGCG-G1 identified (–)-epigallocatechin-3-gallate-4′-*O*-α-D-glucoside as the main compound (CAS 161802-03-5, MW 620.54 g/mol). Its structure is presented in Figure 1. Chemical shifts in ^13^C and ^1^H in DMSO-*d*6 that enabled structure elucidation are presented in Supplementary Materials Figure S1. Due to a novel process including a particular reaction medium composition, high selectivity of the enzyme allowed us to reach (–)-epigallocatechin-3-gallate-4′-*O*-α-D-glucoside purity >70%, compared to previous works where it represented 20 to 40% of total glucosides [29,30]. The remaining 30% of EGCG-G1 are mainly composed of other monoglucosides, and minor diglucosides. 

### 2.2. EGCG-G1 Skin Penetration

**Figure 2:**
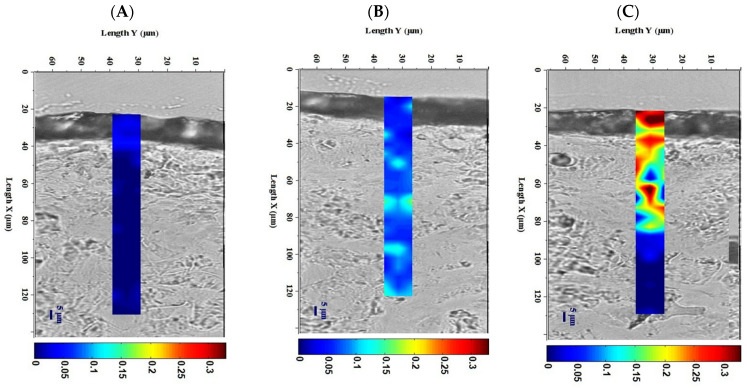
Skin sections and Z-profile analysis using confocal micro-imagery Raman spectroscopy of skin explants with no treatment (**A**), and topically treated with EGCG (**B**) or EGCG-G1 (**C**) at equivalent molarity for 8 h. Depth penetration is indicated by color scale from dark blue (no penetration) to dark red (high penetration) expressed in arbitrary units.

Confocal Raman spectroscopy measured the penetration of EGCG and EGCG-G1 deposited at the surface of skin explants. Both compounds were tested at equivalent molarity. The profiles of the products’ penetration in the epidermis are presented in Figure 2. After 8 h of application, EGCG was slightly detected up to 100 µm depth into the epidermis. EGCG-G1 was detected at a high concentration up to 65 µm, corresponding to an efficient penetration of the glucoside within the stratum corneum, and reached living cells of the epidermis. After semi-quantification of the signal, the amount of EGCG-G1 that penetrated into the skin increased by +90% in comparison to the EGCG (*p* < 0.01 **).

### 2.3. Affinity of EGCG and EGCG-G1 for GLUT1 Transporter

The molecular docking of a protein and its ligand gives an affinity score; the higher the score, the higher the affinity for the protein is. Here, the affinity of α-glucose, EGCG and EGCG-G1 for the glucose transporter GLUT1 was modeled. Indeed, the better penetration of EGCG-G1 in the epidermis suggested a transport made possible by the glucoside moiety. GLUT1 is a glucose transporter highly expressed in keratinocytes and could be a candidate for EGCG-G1 transport. Supplementary Materials’ Figure S2 presents the glucose binding site of GLUT1 and provides details on EGCG and EGCG-G1 interaction with the transporter.

The docking experiment on EGCG and EGCG-G1 with GLUT1 revealed both ligands bound the same cavity with a higher score than glucose. For the inward and outward conformations of the transporter, EGCG presented the bicycle substructure placed at the glucose position according to the crystal structure, while EGCG-G1 presented the glucose moiety at this position. 

Regarding the outward conformation, glucose had a 3.47 score affinity, EGCG 5.11 and EGCG-G1 7.56. In the inward conformation, glucose had a 4.7 score affinity, EGCG 4.87 and EGCG-G1 5.11. The affinity of EGCG-G1 for GLUT1 was higher than that of EGCG according to the models. Both glucoside and aglycone presented a slightly higher affinity than glucose.

### 2.4. In Vitro Antioxidant Activity of EGCG-G1

**Figure 3:**
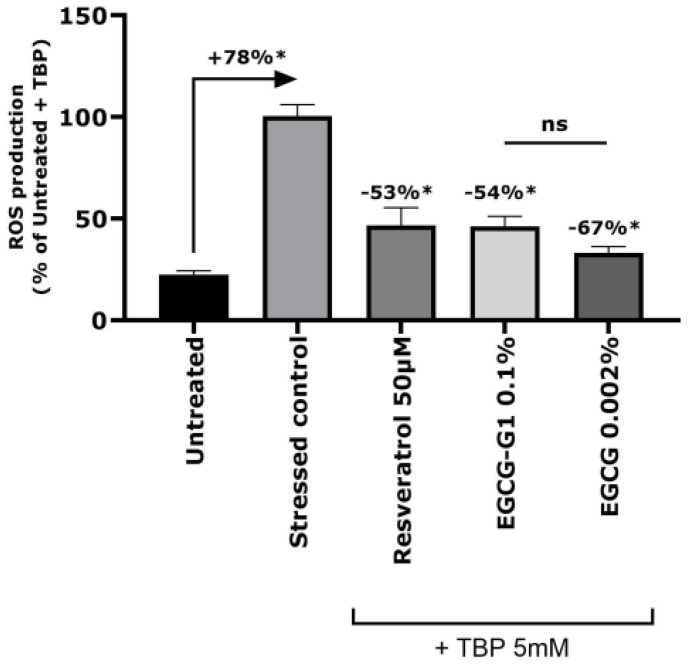
Intracellular Reactive Oxygen Species’ (ROS) production after oxidative stress induction by TBP 5 mM on normal human epidermal keratinocytes pre-treated with resveratrol 50 µM (positive antioxidant control), EGCG-G1 0.1%, EGCG 0.002% (equivalent to EGCG-G1 in molarity) or not (stressed control). ns: not significant, * *p* < 0.05.

The antioxidant effect has been evaluated at the in vitro level on Normal Human Epidermal Kerarinocytes (NHEKs) using the DCFH-DA fluorescent probe to track the intracellular Reactive Oxygen Species’ (ROS) production. Using TBP at 5 mM, cells produced significantly more ROS, up to +78%. Treatment with Resveratrol, a positive antioxidant control, at 50 µM, significantly reduced ROS production by 53%. Meanwhile, EGCG-G1 at 0.1% and EGCG at 0.002% significantly reduced ROS production by −54% and −67%, respectively, in the presence of TBP in comparison to the stressed control (Figure 3).

### 2.5. Impact of EGCG-G1 on Melanogenesis Ex Vivo

**Figure 4:**
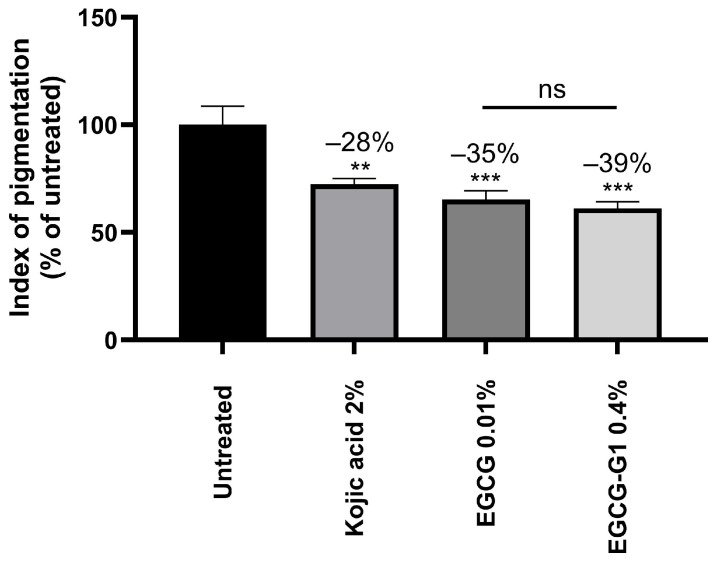
Index of skin explants’ pigmentation following no treatment (untreated), or treatment with kojic acid at 2%, EGCG at 0.01%, or EGCG-G1 at 0.4%, after 5 days of topical treatment. Concentrations of EGCG and EGCG-G1 are equivalent in molarity. ns: not significant, ** *p* < 0.01, *** *p* < 0.001.

Skin explants, from a donor with melanin-rich skin type, received a topical treatment with kojic acid 2% (positive control), EGCG 0.01% or its glucoside form EGCG-G1 at 0.4%, equivalent in molarity to EGCG concentration. The melanin content was significantly reduced by −28%, –35% and −39%, respectively, in comparison to the untreated skin explants (Figure 4). 

### 2.6. Impact of EGCG-G1 on Genes Controlling Melanogenesis

To evaluate the impact of EGCG-G1 on melanogenesis pathways, a co-culture of keratinocytes and melanocytes was established, and a gene expression study was conducted. The results are presented in Table 1. After 24 h of treatment with EGCG-G1 at 0.01%, the RT-qPCR revealed that the glucoside was able to significantly reduced the expression of genes involved in different signaling pathways of melanogenesis: KIT (Tyrosine Kinase) –93%, MC1R (Melanocortin 1 receptor) –96%, EDNRB (Endothelin receptor type B) –68%, SOX10 (Sex-determining region Y-box 10) –60%, and MITF (Microphthalmia-associated Transcription Factor) –108%. KIT is expressed in keratinocytes and fibroblasts contributing to paracrine regulation of melanogenesis with its ligand SCF (Stem cell factor). SCF/KIT is considered a main pathway of melanogenesis [1] with MC1R/α-MSH (melanocortin-stimulating hormone). EDNRB is expressed in keratinocytes that produce endothelin 1 (EDN1): the binding of EDN1 to EDNRB triggers a complex signaling cascade that leads, as in the previous pathways, to MITF activation. MITF is a pivotal transcription factor of melanogenesis that regulates itself by the SOX10 transcription factor. 

EGCG-G1 was also able to significantly reduced gene expression of melanogenic enzymes TYR (Tyrosinase) –58%, and TYRP1 (Tyrosinase-related protein 1) –95%, which directly contribute to the synthesis of melanin. 

The glucoside down-regulated several genes involved in melanosome biogenesis as follows: AP3B1 (Adaptor-related protein complex 3 subunit beta 1) –59%, GPR143 (G protein-coupled Receptor 143) –365%, PMEL (Melanocyte specific protein) –212%, DTNBP1 (Dystrobrevin Binding Protein 1) –59% and LYST (Lysosomal Trafficking regulator) –48%. The function of the related proteins has been less studied and not fully characterized until today. AP3B1 controls melanin production and tyrosinase distribution in melanosome [32]. GPR143 regulates melanosome size and trafficking [33]. PMEL produces sheet fibrils inside melanosomes that contribute to melanin synthesis and transport [34]. DTNBP1 is a subunit of BLOC1 (Biogenesis of Lysosomal Organelles complex-1) and participates in the transfer enzymes from endosome to melanosome [35].

F2RL1 (F2R like trypsin receptor 1), also known as PAR2 (protease-activated receptor 2), increases phagocytosis of melanosomes by keratinocytes and the corresponding gene was also down-regulated by EGCG-G1 (−49%) compared to the untreated co-culture [36]. 

### 2.7. Impact of EGCG-G1 on Bacterial Metabolism

#### 2.7.1. EGCG-G1 Intake by *Bacillus* sp.

**Figure 5:**
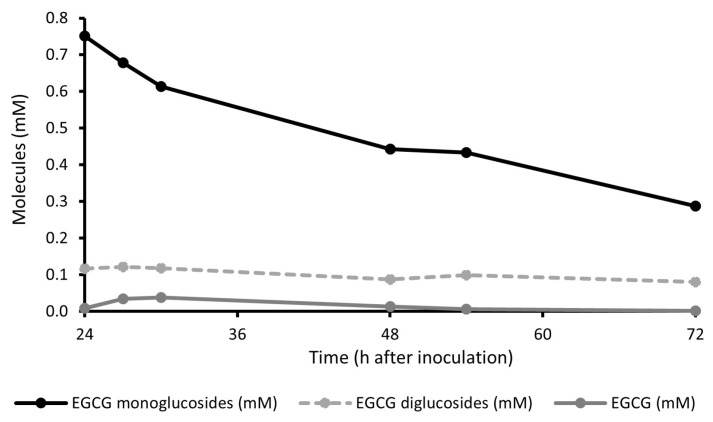
Quantity of EGCG, EGCG mono- and diglucosides in culture medium of *Bacillus* sp. isolated from normal skin. Bacteria was grown for 24 h before adding the active.

*Bacillus* sp. GAB 04-01, isolated from normal skin, was precultured for 24 h and then EGCG-G1 was added to bacterial culture. HPLC analysis followed EGCG and derivatives evolution in the culture medium (Figure 5). The residues of EGCG and EGCG diglucosides remained stable, while the concentration of EGCG monoglucosides decreased by 2.6-fold. The stability of EGCG indicated that monoglucosides were not converted in EGCG by hydrolysis of glucose moiety. No increase in other EGCG derivatives was detected. These data suggest that EGCG-G1 monoglucosides are not degraded by the bacteria but assimilated by the micro-organism. 

#### 2.7.2. EGCG-G1 Impact on *Lactobacillus* sp. Growth and Metabolism 

**Figure 6:**
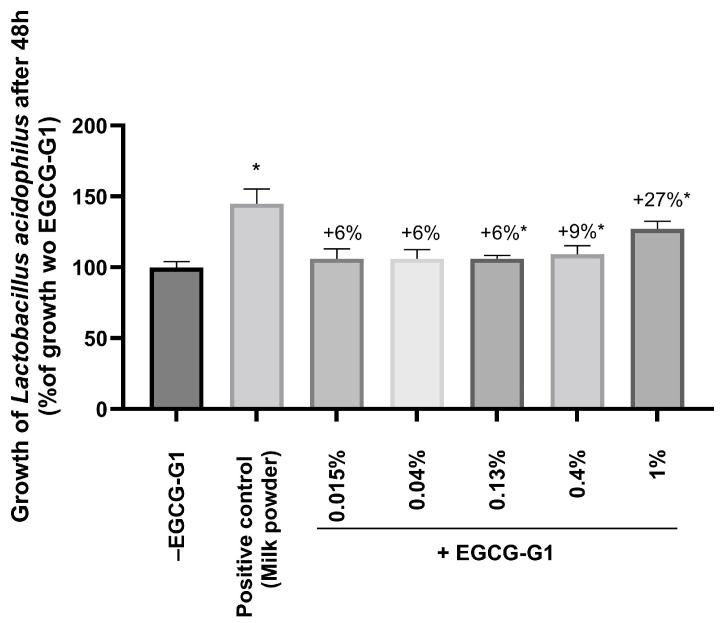
Growth of *Lactobacillus acidophilus* analyzed using OD600 nm after 48 h of culture. Bacteria were cultivated without EGCG-G1 (−EGCG-G1), with milk powder as a positive control and with several concentrations of EGCG-G1 (+EGCG-G1) from 0.015% to 1%. The results are expressed in percentage of growth without EGCG-G1. * *p* < 0.05.

It has recently been established that *Lactobacillus* sp. are inhabitants of skin surface and produce metabolites with antioxidant and anti-melanogenic properties [37]. To further the understanding of EGCG-G1 impact on microbiota, the growth of *Lactobacillus acidophilus* in MRS medium supplemented or not with several concentrations of EGCG-G1 was observed. Bacterial growth was measured using the optical density of the culture at 600 nm. Milk powder at 0.01% was used as the positive control and significantly increased the growth of the bacteria, validating the experiment. Regarding EGCG-G1, the active was able to significantly increase the growth of *L. acidophilus* with a dose–response effect by +6%, +9% and +27%, at 0.13%, 0.4% and 1% concentration, respectively, in comparison to the culture without EGCG-G1 (Figure 6).

**Figure 7:**
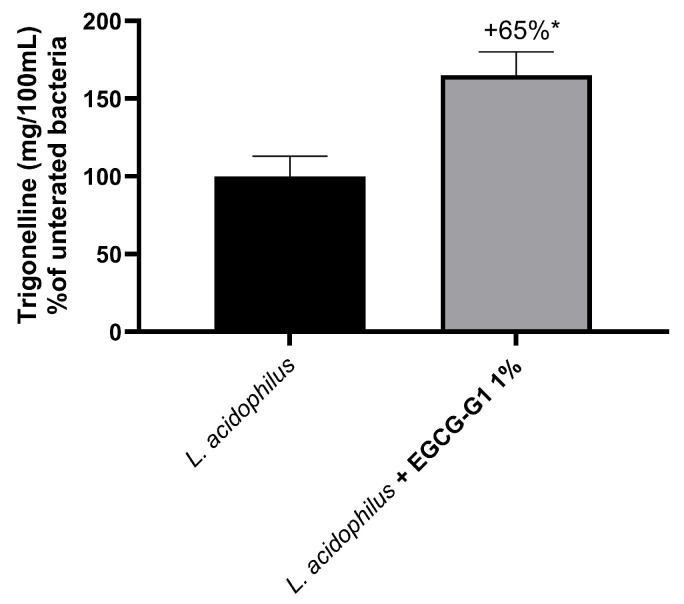
Quantification of trigonelline by high-field NMR in culture medium of *L. acidophilus* supplemented with EGCG-G1 1% relative to untreated condition. * *p* < 0.05.

Metabolites of the culture medium containing EGCG-G1 1% were analyzed by high-field NMR and compared to the untreated culture. Firstly, culture with EGCG-G1 1% changed the carbon source of the strain for its metabolism. Treatment with 1% EGCG-G1 decreased sugars (glucose, xylose) uptake by −17% (*p* = 0.1), not significantly, in favor of fatty acids (polysorbate 80) consumption, which significantly increased by +51% (*p* < 0.05). Another noticeable change induced by EGCG-G1 was the increase in trigonelline (+65%) in the culture medium of *L. acidophilus* (identified by signal at 9.11 ppm), as presented in Figure 7. It suggested that trigonelline is synthesized and released from bacteria due to the presence EGCG-G1.

### 2.8. Clinical Evaluation of EGCG-G1 on Skin Hyperpigmentation and Microbiota

Four double-blinded, placebo-controlled studies were conducted for 56 days to address several questions. Clinical trials were designed with volunteers of 18 to 75 years old, covering phototypes I to V, and targeting face and hands hyperpigmentation disorders. EGCG-G1 was benchmarked against EGCG or vitamin C in two studies. Several tools dedicated to skin tone and color evaluation were used. Parameters and analysis have been summarized in the Table 2 below.

#### 2.8.1. EGCG-G1 Reduced Uneven Skin Tone and Hyperpigmented Spots in Facial Asian Mature Skin Better than Vitamin C

**Figure 8:**
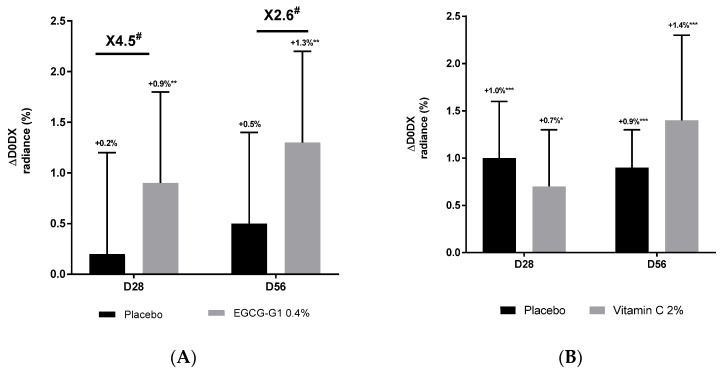
Impact of EGCG-G1 0.4% (**A**) and vitamin C 2% (**B**) on radiance of mature Asian skin measured by Colorface^®^ at 28 and 56 days of application compared to day 0. # *p* < 0.1, * *p* < 0.05, ** *p* < 0.01 and *** *p* < 0.001.

**Figure 9:**
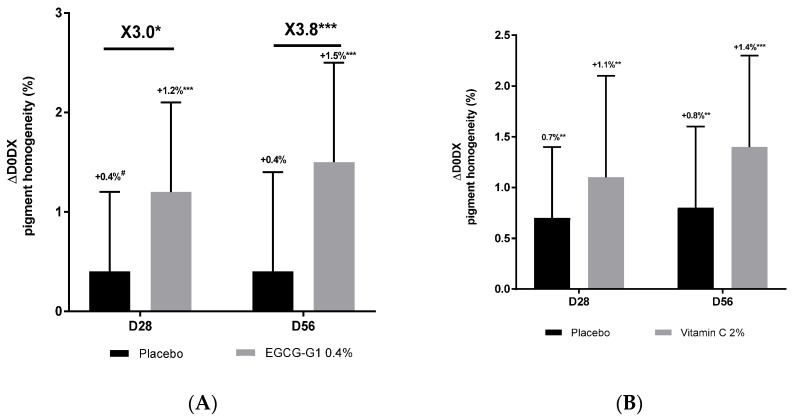
Impact of EGCG-G1 0.4% (**A**) and vitamin C 2% (**B**) on pigmentation homogeneity of mature Asian skin measured by Colorface^®^. # *p* < 0.1, * *p* < 0.05, ** *p* < 0.01, and *** *p* < 0.001.

**Figure 10:**
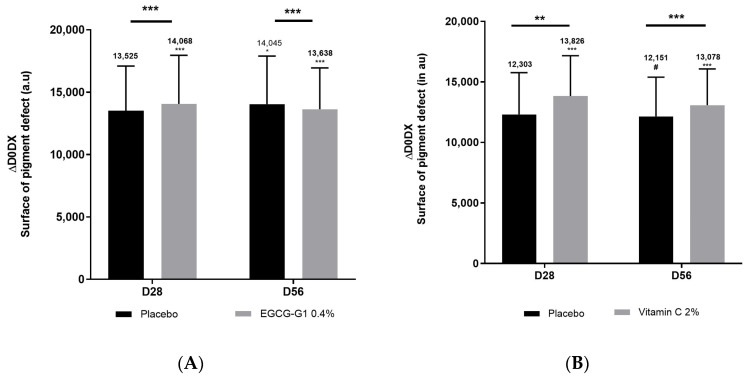
Impact of EGCG-G1 0.4% (**A**) and vitamin C 2% (**B**) on hyperpigmented spots’ surface of mature Asian skin measured by Colorface^®^. # *p* < 0.1, * *p* < 0.05, ** *p* < 0.01 and *** *p* < 0.001.

A clinical trial dedicated to mature Asian skin was conducted. Inclusion criteria targeted women of 45 to 75 years old with hyperpigmented spots on face. One group applied EGCG-G1 0.4% or not (placebo) on the hemiface, and the other applied vehicle supplemented with vitamin C 2% (benchmark) or not (placebo) on the hemiface too. Colorface^®^ was used to measure radiance (Figure 8), skin homogeneity (Figure 9) and hyperpigmented spots through the measure of surface covered by pigment defects (Figure 10). Compared to day 0, EGCG-G1 progressively increased radiance up to +1.3% and pigment homogeneity up to +1.5%. It decreased hyperpigmented spots by −16.9%. EGCG-G1 specifically decreased hyperpigmented spots’ visibility but also improved the overall skin face glow. Illustrative pictures are presented in Supplementary Materials Figure S3. Similar absolute values were recorded for vitamin C. Nevertheless, comparison with placebo demonstrated that EGCG-G1 was more efficient than vitamin C: as an example, at day 56, EGCG-G1 decreased hyperpigmented spots by −21.7% vs. placebo, while vitamin C decreased pigmentation defects at −13.9% vs. placebo (Figure 10). 

#### 2.8.2. EGCG-G1 Reduced Brown Spots and Increase Facial Skin Lightness on African Skin and Outperformed EGCG

**Figure 11:**
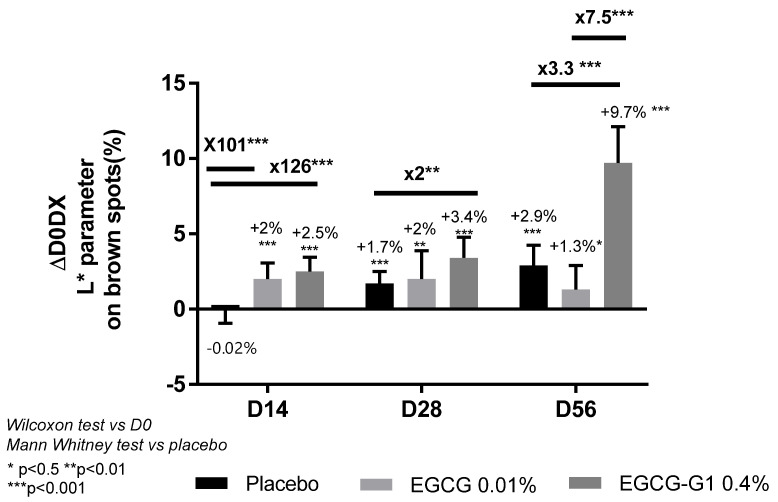
Impact of EGCG-G1 0.4%, EGCG 0.01% (equivalent in molarity to EGCG-G1 0.4%) or placebo on L* parameter (lightness) of brown spots. At days 14, 28 and 56 of application compared to day 0. * *p* < 0.05, ** *p* < 0.01, and *** *p* < 0.001.

A clinical trial on African skin was conducted. The inclusion criteria targeted 85 women from 18 to 47 years old, with dull skin, hyperpigmentation and skin heterogeneity. One group applied EGCG-G1 0.4%, the second group applied vehicle supplemented with EGCG 0.1%, equivalent to ECGC-G1 in molarity, and the third group applied vehicle only (placebo) on the whole face. A Chromameter^®^ was used to measure L* parameter on brown spots area (Figure 11). As a reminder, L* is lightness and it is evaluated from 0, black, to 100%, white. A reduction in skin pigmentation is associated with an increase in the L* parameter. A significant increase in the L* parameter was observed on brown spots of women applying EGCG-G1 0.4% vs. D0 and vs. placebo: +2.5% on D14, +3.4% on D28 and +9.7% on D56. Illustrative pictures are presented in Supplementary Materials Figure S4 EGCG also increased the lightness of brown spots by +2% on D14 and D28 but was not significant vs. placebo on day 56. EGCG-G1 clearly outperformed the aglycone in hyperpigmented spots’ blurring.

**Figure 12:**
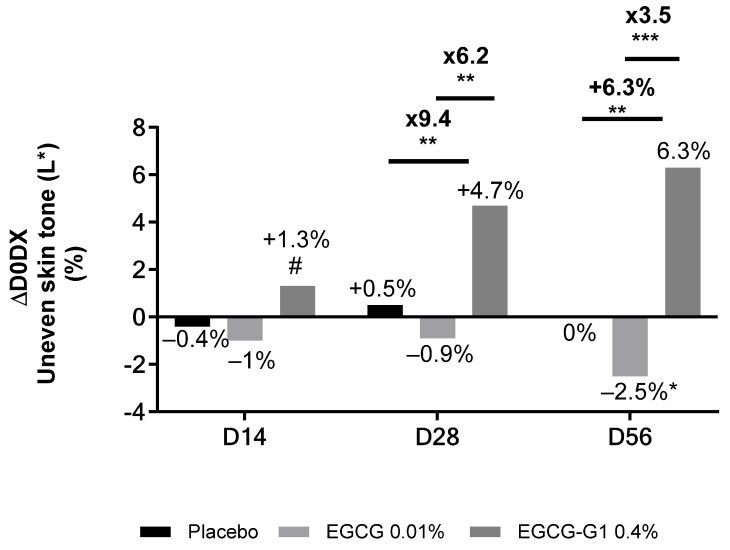
Impact of EGCG-G1 0.4% versus EGCG 0.01% (equivalent in molarity to EGCG-G1 0.4%) or placebo on skin tone from African skin type by using L* parameter compared to day 0. # *p* < 0.1, * *p* < 0.05, ** *p* < 0.01, and *** *p* < 0.001.

Then, whole-skin face homogeneity was evaluated by measuring the overall L* parameter (Figure 12). A progressive and significant increase in the L* parameter was observed with EGCG-G1 0.4%: +1.3% on D14, +4.7% on D28 and +6.3% on D56. The aglycone displayed no significant effect on the L* parameter except on D56, where a decrease was observed, suggesting EGCG increased skin heterogeneity. This could be related to a light brightening effect on brown spots observed in Figure 11, with no improvement in skin tone that led to higher heterogeneity. 

#### 2.8.3. EGCG-G1 Reduced Brown Spots and Increased Facial Skin Lightness on Indian Skin 

A clinical trial on Indian skin was conducted. The inclusion criteria targeted 38 women of 18 to 30 years with melanin-rich skin. One group applied EGCG-G1 0.4% and the other applied placebo on the whole face. Skin color was analyzed by Chromameter^®^ and Visioface^®^ methodologies.

A Chromameter^®^ was used to measure the L* parameter, ITA and ΔE. ITA determines the individual typology angle that describes skin color, and the higher the ITA, the lighter the skin. ΔE evaluates color differences between skin sites, giving a value representative of skin heterogeneity; here, the same six skin sites were evaluated along the 56 days of study and the lower the ΔE, the higher homogeneity. 

**Figure 13:**
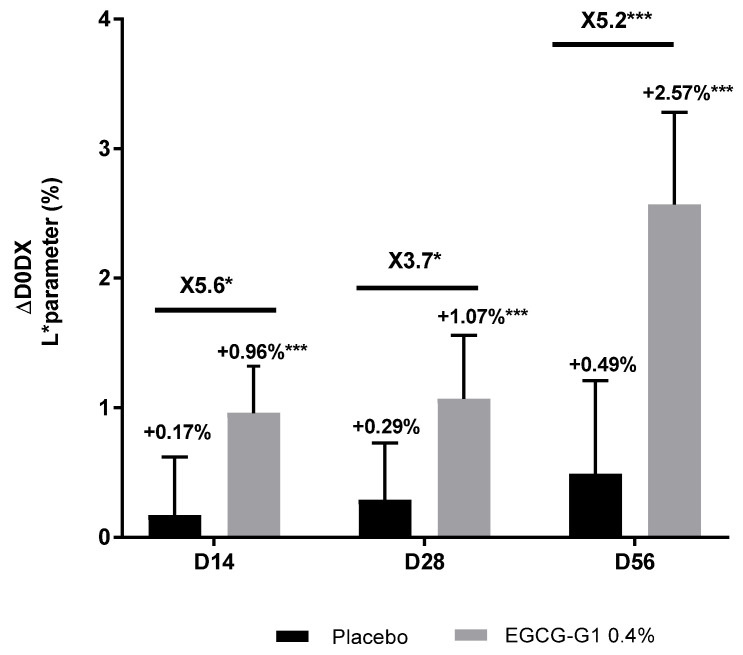
Impact of EGCG-G1 at 0.4% versus placebo on color of Indian skin type evaluated by measuring the L* parameter with a Chromameter^®^. * *p* < 0.05 and *** *p* < 0.001.

A significant and progressive brightening effect of EGCG-G1 was observed by the increase in the L* parameter by +0.96% on D14, +1.07% on D28 and + 2.57% on D56 compared to D0 (Figure 13). Illustrative pictures are presented in Supplementary Materials Figure S5. Compared to placebo, EGCG-G1 showed an L* increase by ×5.6-fold on D14, ×3.7 on D28 and ×5.2 on D56.

**Figure 14:**
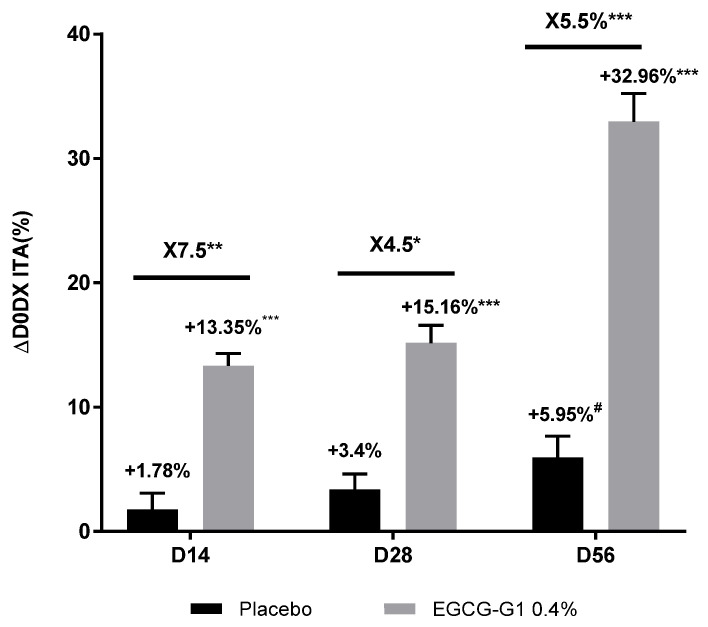
Impact of EGCG-G1 at 0.4% versus placebo on color of Indian skin type by ITA measurement using Chromameter^®^ # *p* < 0.1, * *p* < 0.05, ** *p* < 0.01, and *** *p* < 0.001.

A significant and progressive brightening effect was confirmed by the increase in ITA by +13.35%, +15.16% and +32.96% on D14, D28 and D56, respectively, compared to D0 (Figure 14). A significant and better effect was observed compared to placebo, showing ×7.5, ×4.5 and ×5.5 increase in brightening performance on D14, D28 and D56, respectively.

**Figure 15:**
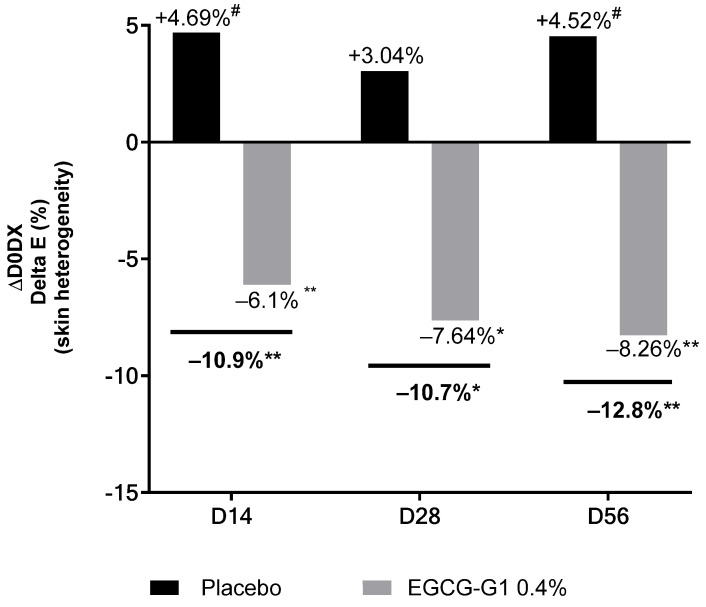
Impact of EGCG-G1 at 0.4% versus placebo on color of Indian skin type evaluated by ∆E value expressing skin heterogeneity and measured by Chromameter^®^. # *p* < 0.1, * *p* < 0.05 and ** *p* < 0.01.

Skin heterogeneity, evaluated by ΔE measurement, decreased in the group treated with EGCG-G1 by −6.1% on D14, by −7.64% on D28 and by −8.26% on D56, compared to D0 (Figure 15). These results were also significant compared to placebo: ΔE decreased by −10.9% on D14, by −10.7% on D28 and by −12.8% on D56. 

**Figure 16:**
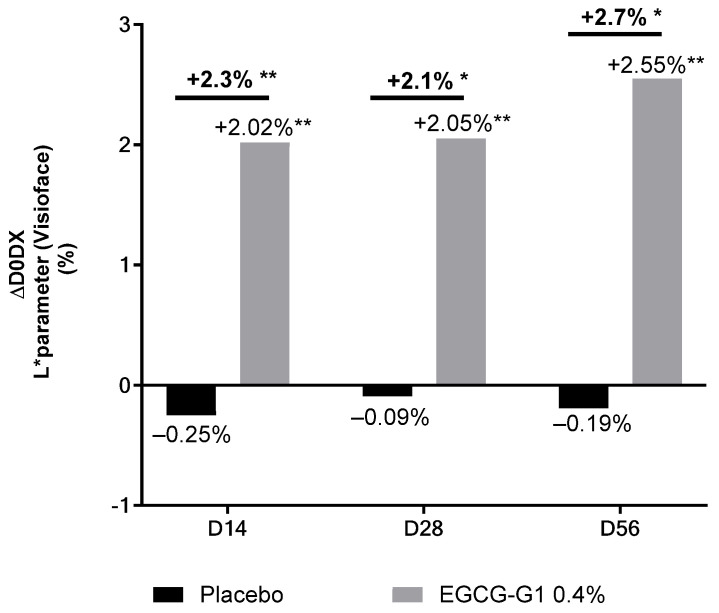
Impact of EGCG-G1 at 0.4% versus placebo on color of Indian skin type by measuring L* parameter using Visioface^®^. * *p* < 0.05 and ** *p* < 0.01.

**Figure 17:**
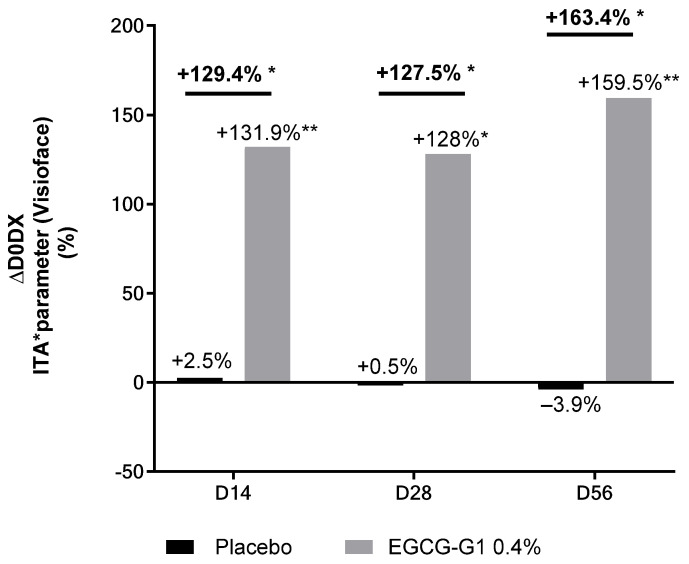
Impact of active at 0.4% versus placebo skin color from Indian skin type by ITA using Visioface^®^. * *p* < 0.05 and ** *p* < 0.01.

Visioface^®^ methodology was used to give full face analysis of skin lightening. Again, the L* parameter (Figure 16) and ITA (Figure 17) were measured. A significant and progressive brightening effect was observed by the increase in L* value by +2.02%, +2.05% and + 2.55% on D14, D28 and D56, respectively. A significant and better brightening effect, compared to placebo, was also observed by the increase in L* by +2.3%, +2.1% and + 2.7% on D14, D28 and D56, respectively.

A significant and progressive brightening effect was confirmed by the increase in ITA value by +131.9%, +128% and +159.5% on D14, D28 and D56, respectively, in the group treated with 0.4% EGCG-G1 compared to D0. Compared to placebo, EGCG-G1 also significantly increased ITA by +129.4%, +127.5% and +163.4% on D14, D28 and D56, respectively.

#### 2.8.4. EGCG-G1 Reduced Brown Spots on Fair Skin Hands 

**Figure 18:**
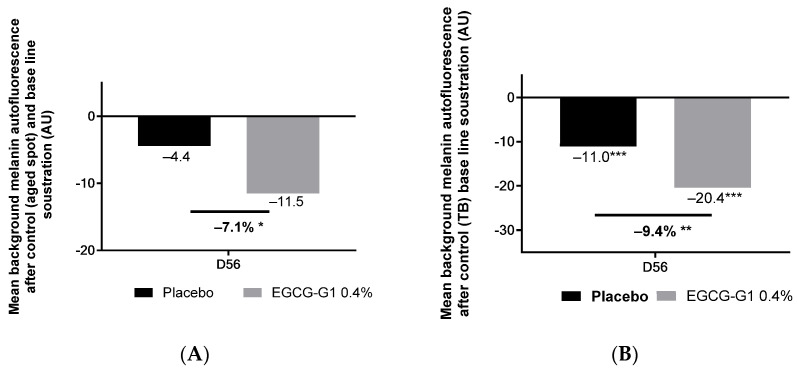
Impact of EGCG-G1 at 0.4% versus placebo on melanin content using autofluorescence detection by Raman spectroscopy after 56 days of application (**A**); and on skin heterogeneity using melanin autofluorescence detection from pigmented and unpigmented zones by Raman spectroscopy after 56 days of application (**B**). * *p* < 0.05, ** *p* < 0.01, and *** *p* < 0.001.

Finally, a clinical trial was conducted on the hands of mature Caucasian skin. The inclusion criteria targeted 30 women from 45 to 75 years old presenting hyperpigmented spots on hands. Indeed, hands are a sun-exposed surface, where solar lentigo develops [38]. While these are benign lesions, they represent a beauty defect and a sign of aging. EGCG-G1 0.4% formulated in cream was applied by one group for 56 days and the other group applied placebo. Skin color was analyzed by Raman spectroscopy analysis by using autofluorescence of the melanin (Figure 18). Two different measures were performed: in Figure 18A, melanin content was determined on brown spots, and in Figure 18B, melanin was detected on the spotted area versus skin baseline to evaluate skin heterogeneity. 

Regarding melanin autofluorescence measurement on brown spots, the valued decreased by −11.5 AU in the group treated with EGCG-G1 and by −4.4 in the group applying placebo, compared to D0. Illustrative pictures are presented in Supplementary Materials Figure S6. When compared to placebo, EGCG-G1 reduced melanin autofluorescence by −7.1%, suggesting EGCG-G1 is able to decrease pigment concentration in these hyperpigmented spots.

Considering skin heterogeneity, it significantly decreased by −20.4 AU in the group applying EGCG-G1 on hands and by −11.0 in the group treated with placebo, compared to D0. When comparing EGCG-G1 to placebo, the glucoside reduced skin heterogeneity by −9.4%.

#### 2.8.5. EGCG-G1 Increased Cheek Skin Lactobacillus Population In Vivo 

**Figure 19:**
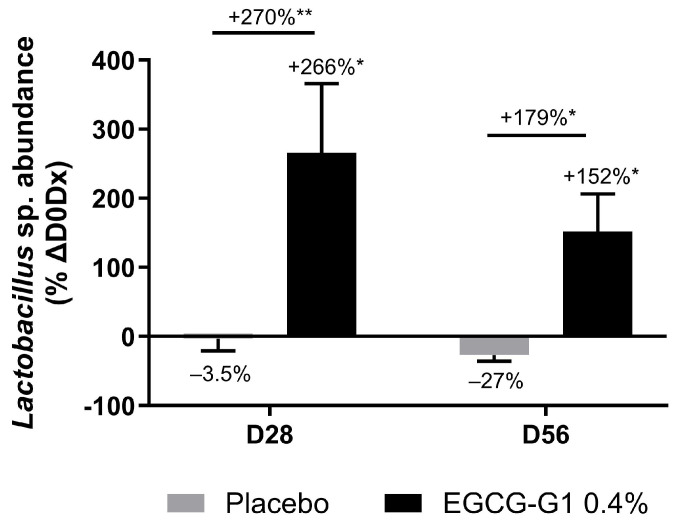
*Lactobacillus* relative abundance evolution after 28 and 56 days of application of EGCG-G1 0.4% cream and placebo on face of volunteers. * *p* < 0.05, ** *p* < 0.01.

The clinical trial on white hands presented in Section 2.8.4 led us to be interested in the skin microbiota of cheeks. After microbiota sampling, 16S rRNA sequencing allowed us to determine the evolution of bacterial community composition following placebo or EGCG-G1 0.4% application. After 28 and 56 days of treatment, neither the placebo nor the active product modified the diversity of cheek skin microbiota (Shannon index analysis). EGCG-G1 did not impact microbiota composition at the phylum level but a significant increase in the genus *Lactobacillus* was observed (Figure 19).

## 3. Discussion

Melanin and more particularly eumelanin protect skin from deleterious effects of UVR [10]. Nevertheless, UVR induces skin photo-aging associated with hyperpigmentation disorders affecting all skin phototypes [8,9,12,39]. 

EGCG is a natural phenolic compound highly concentrated in green tea, attracting attention of researchers in the treatment of cancer and photocarcinogenesis [23,40]. EGCG displays skin photoprotective properties: thickening of epidermis and barrier function improvement; antioxidant activity decreasing UV-induced ROS, lipid peroxidation, DNA damage, inflammation, metalloproteinases and apoptosis; and increasing antioxidant enzyme activity [24,41,42]. EGCG also promotes skin hydration and decreases melanogenesis under UVR exposure [24]. EGCG reduces melanin content by decreasing MITF and TYR proteins and slows down melanosome maturation [25,43,44]. The MC1R signaling pathway is down-regulated by EGCG [45] but little is known about the impact of the phenolic compound on the other pathways regulating melanogenesis. Anti-melanogenic activity of EGCG is stronger than reference compounds kojic acid and arbutin; so, it is an interesting candidate in the control of hyperpigmentation disorders combining photoprotective effects. However, this phenolic compound is poorly water-soluble, presents low stability in aqueous solution and low skin permeability, limiting its use for cosmetic application [28,46].

Enzymatic glucosylation of EGCG enhances the stability and water solubility of the native molecule [28]. In this work, a monoglucoside called EGCG-G1, identified as ()-epigallocatechin-3-gallate-4′-O-α-D-glucoside, was obtained at high purity (>70%) compared to previous reports [29,30]. Enhanced water solubility and stability of EGCG-G1 were confirmed (data not shown). Glucosylation did not modify the antioxidant activity of EGCG-G1 compared to EGCG, suggesting the glucoside retained the photoprotective effects of the aglycone described above. UVR-induced oxidative stress also promotes melanogenesis through different pathways [47]; so, phenolic antioxidants as EGCG-G1 generally inhibit melanin synthesis associated with sun exposure.

Interestingly, we demonstrated that glucosylation greatly improved EGCG-G1 skin permeability (+90% vs. EGCG) up to 65 µm, meaning EGCG-G1 is able to cross stratum corneum and reach living cells of the epidermis. It can unlikely be attributed to glucosylation, which confers higher hydrophilicity, and thus EGCG-G1 should be less prone to interact with skin lipids. It was also hypothesized that EGCG-G1 could be internalized by the glucose transporter GLUT1, highly expressed in keratinocytes [48]. Molecular docking indicated EGCG-G1 can bind GLUT1 in the same pocket as glucose with higher affinity than EGCG and the carbohydrate; so, this hypothesis could be further explored. 

EGCG-G1 antimelanogenic activity was confirmed on skin explants and was equivalent to EGCG in this model. Co-culture of keratinocytes and melanocytes was used to study the impact of EGCG-G1 on 13 genes involved in melanogenesis [1]. MC1R, KIT and EDNRB were down-regulated, confirming previous findings that the signaling pathway α-MSH/MC1R is modulated by EGCG, and we showed that the SCF/KIT, EDN1/EDNRB pathways are also impacted by EGCG-G1. The gene expression of the pivotal transcription factor MITF was also decreased by EGCG-G1, together with SOX10, a transcription factor controlling MITF. EGCG-G1 decreased the expression of melanogenic enzymes TYR and TYRP1, directly affecting melanin synthesis. The phenol glucoside also strongly decreased the expression of genes encoding proteins necessary to melanosome biogenesis, especially PMEL and GPR143, and of F2RL1, playing a role in melanin phagocytosis by keratinocytes. PMEL protein is induced by UVR exposure under MITF control. PMEL forms fibrils within melanosome, where melanin is deposited and could contribute to melanin polymerization. GPR143 is a G-protein-coupled receptor for melanin precursors, localized in the melanosome membrane and involved in the control of melanosome size, composition and maturation. 

Our laboratory recently published data on regulators of solar lentigo and brown spots development [19,38]. Our results revealed a specific microbiota composition and networks on skins based on hyperpigmented spots status. As an essential part of the overall skin ecosystem, and through its interaction with human cells, microbiota could be considered a new target for skincare applications. So, a clinical study was conducted to evaluate the effect of EGCG-G1 on skin microbiota. After 28 days of application, EGCG-G1 increased *Lactobacillus* population on cheek skin. A previous study suggested *Lactobacillus,* as a part of the skin microbiota, may develop protective effects from photoaging [49,50]. *Lactobacillus* is also used as a probiotic to improve skin hydration and elasticity and decrease pigmentation [51,52]. In addition, lipoteichoic acid and lactic acid, two metabolites of lactic bacteria, inhibit melanogenesis and matrix-degrading enzymes [20,53]. 

That is why EGCG-G1 was applied to *Lactobacillus acidophilus* culture as a model to identify metabolites changes compared to the untreated culture. EGCG-G1 increased bacterial growth, as observed in skin microbiota. EGCG was shown to increase the viability of several *Lactobacillus* species while exhibiting an antimicrobial effect on pathogenic bacteria [54]. Glucosylation generally decreased cell toxicity [28]; so, EGCG-G1 could be even more favorable to lactic bacteria than EGCG. 

In the presence of EGCG-G1, we observed an increase in oleic acid metabolism in the form of polysorbate 80 (data not shown). We previously observed a possible interaction between glucose carrier GLUT1 and EGCG-G1 with a score affinity higher than that of glucose. Bacteria have a structural analog of this carrier, named XylE, with a global homology of 62% in comparison to GLUT1 and 75% of the binding site was conserved between the species [55]. EGCG-G1 might be internalized in bacteria using XylE carrier instead of sugars, thus explaining the higher uptake of fatty acids. 

Another noticeable change was a higher content of trigonelline in the medium supplemented with EGCG-G1, suggesting *L. acidophilus* synthesized a greater amount of this molecule. Trigonelline, a methylated form of niacin, has been reported as result of the lactic bacteria fermentation [56]. Recently trigonelline was suggested to have tyrosinase inhibitory effect [57]. Further studies are necessary to explain the link between *Lactobacillus* and EGCG-G1 and melanogenesis.

Finally, four clinical trials were conducted to evaluate the effect of EGCG-G1 on skin pigmentation disorders of women with different ancestry (Asian, African, Indian and Caucasian). Genetic background is responsible for constitutive pigmentation and UVR induce differential melanogenic responses depending on skin types [9]. A limitation of trials design was the use of several devices due to technical constraints, that restrict the comparison of EGCG-G1 benefits on the different phototypes. Nevertheless, in all clinical studies, EGCG-G1 progressively and significantly increased skin lightness and homogeneity and decreased localized hyperpigmentation vs. D0 and placebo, whatever the phototype, age, or device used. 

EGCG-G1 was benchmarked against vitamin C (ascorbic acid) used in the treatment of skin hyperpigmented spots [58]. Vitamin C inhibit TYR by interacting with copper ions at the active site of the enzyme has also antioxidant activity. Both compounds improved radiance, pigmentation homogeneity and decreased surface pigment defect compared to D0. Nevertheless, the difference between placebo and the product was greater for EGCG-G1, suggesting a better efficacy of the phenolic glucoside vs. placebo. 

EGCG-G1 was also evaluated vs. EGCG at similar molarity. It is interesting to reiterate that both compounds triggered similar melanin content reduction on phototype V skin explants. In the clinical trial, conducted on volunteers with African ancestry, the aglycone had a significant effect on brown spots’ L* parameter (+2%) at D14 and D28 that was not observed on D56, while EGCG-G1 progressively increased L* to +9.7% on D56. The slight effect of EGCG was probably too low to trigger a visible effect on full face measurement, and no significant improvement was detected on skin homogeneity and skin brightness. Glucosylation generally alters the bioactivity of flavonoids but certain types of biological benefits are enhanced [59], as is the case for the brightening properties of EGCG-G1. 

## 4. Materials and Methods

### 4.1. EGCG-G1 Obtention and Characterization

EGCG-G1 was prepared from EGCG according to the international patent application WO 2024/100140. Briefly, EGCG-G1 was obtained by a cell-free process, where saccharose was the glucose donor, and transfer was achieved by a glucansucrase produced in our laboratory. The reaction mixture was optimized to enhanced enzyme selectivity. 

EGCG-G1 was purified by centrifugal partition chromatography, as previously described [60], and fractions containing the main monoglucoside were grouped, evaporated to dryness and suspended in DMSO-*d*6 before ^13^C, HSQC, HMBC and COSY analyses. 

### 4.2. EGCG-G1 and EGCG Concentrations 

Concentrations of molecules are presented along this article in percentage; these are usually used to express active content in cosmetic products. In order to achieve a fair comparison between EGCG (458.37 g/mol, pure powder) and EGCG-G1 (638.51 g/mol, diluted in solvent), the compounds were applied at the same molarity in each test, corresponding to 0.002% of EGCG and 0.1% of EGCG-G1; 0.01% of EGCG and 0.4% of EGCG-G1; or 0.05% of ECGC and 2% of EGCG-G1. 

### 4.3. Comparison of EGCG and EGCG-G1 Skin Penetration Using Raman Spectroscopy

#### 4.3.1. Skin Explants’ Culture and Preparation

Human skin explants coming from a 35-year-old donor who had had an abdominoplasty were prepared and kept in survival medium (MIL215001, Biopredic, Saint Grégoire, France) for 24 h at 37 °C and 5% CO_2_. The next day, EGCG at 0.05% and EGCG-G1 2% in sterile water were topically applied and incubated for 8 h at 37 °C and 5% CO_2_ before skin penetration analysis. The untreated condition did not receive any treatment. After the end of incubation, the skin surface was cleaned in order to eliminate any excess of the product. The skin explants were then frozen at −80 °C and cut longitudinally using a cryotome with a thickness of 20 µm. For each explant, 3 tissue sections were selected and deposited on a CaF_2_ support for Raman imaging analysis for a total of 3 Raman images per condition. Four other adjacent sections of 7 µm thickness were prepared for Hematoxylin & Eosin staining.

#### 4.3.2. Raman Micro-Imaging

Raman images have a size of Y: 10 μm/X: 100 μm with a step of 5 μm in X and 5 μm in Y. Each Raman image has 3Y spectra and 22X spectra (66 spectra per image). Images were obtained using the following parameters: laser wavelength at 660 nm; objective 100 X with long focal length at numerical aperture 0.75; acquisition time of 30 s; accumulation 1X; spectral range between 600 and 31,000 cm^−1^; grafting 950T; confocal hole at 300 µm; slide width of 150 µm with spectral resolution of 6.5 cm^−1^; and step in X/Y of 5 µm. In order to ensure reproducibility of the measurements, before each use, the Raman spectrometer is calibrated with silicon, which gives a Raman peak at 520.7 cm^−1^. Continuous control of the laser power at the sample level is achieved. A pre-processing of Raman images was made by eliminating aberrant spectra (fluorescence, burning, saturation), correcting the baseline, applying a spectral smoothing and despike and a spectral normalization. The processing of corrected data maps was performed by using homemade software based on the least squares fitting method that operates with Matlab software (Version R2015a). This method involves mathematical modeling of reference spectra in the overall spectral image to determine the contribution and distribution of these spectra within the image. In this study, we used as reference spectra the one of the respective product (EGCG and EGCG-G1).

### 4.4. Molecular Docking Analysis of EGCG-G1 with GLUT1

#### 4.4.1. Modeling of Inward Conformation of GLUT1

Five structures of the Human glucose transporter GLUT1, all obtained using X-ray diffraction, are available from the Protein Data Bank (PDB) (https://www.rcsb.org accessed on 19 January 2023). All these structures are in an inward conformation with the extracellular domain closed and the intracellular domain opened. They all are very similar, which is shown by an RMSD of 0.6 Å for the backbone and 1.76 Å for the entire protein. On these different structures, the binding site is relatively conserved, and it is composed of the residues: THR137, GLN282, GLN283, ASN288, ASN317, GLU380, TRP388 and ASN411. The different structures were used to build a model for the docking. 

#### 4.4.2. Modeling of Outward Conformation of GLUT1

Currently, no structure of the GLUT1 outward conformation is available, nor is a structure binding glucose. Other structures of Human GLUTs are available in the PDB. Of interest, the GLUT3 member had been crystallized in the outward conformation. The primary sequences of GLUTs are relatively conserved, and the glucose binding site is extremely conserved among GLUTs. Considering the homology of GLUT1 and GLUT3, the outward conformation of GLUT1 was modeled using the structures of the GLUT3 receptor. These structures display backbone RMSD on the full protein of 0.43 Å. On these different structures, the binding site is relatively conserved, and it is composed of the residues: GLN181, GLN302, GLN303, ASN308, ASN337, GLU380 and TRP388.

The different structures were used to build a model for the docking. For each structure, the molecular modeling was performed with SYBYL (2.1.2). At first, GLUT1 and GLUT3 primary sequences were aligned with the module Fugue that takes into account the amino-acid and their local geometry. Then, the Orchestrar module of SYBYL was used to generate homology models of GLUT1 outward conformation based on GLUT3 structures. 

#### 4.4.3. Molecular Docking

The 3D structures for each molecule were generated using the Sybyl tool, Concord, with default parameters. Molecular docking was performed using Surflex-Dock present in Sybyl. To assess the docking and select the best model, multiple RX structures were docked. For the inward conformation, all available crystal structures were considered (PDB ID: 4pyp, 5eqh, 5eqi, 5eqi and 6tha). For the outward conformation, however, the generated model built from GLUT3 crystal structures were considered (PDB ID: 4zw9, 4zwb, 4zwc, 7crz, 7sps and 7spt). We used a combination of analyses to assess the validity of the docking: on one hand, the analysis of crystal structure ligands (glucose, BNG, inhibitors, …) poses, and on the other hand, the analysis of the hit rate of the Top 1%, 5% and 10% compounds with the highest score. After analyzing the different results, the structure 5eqh for the inward conformation and the model based on GLUT3 4zwc for the outward conformation were selected for the docking study of the EGCG and EGCG-G1 molecules. Though 5eqi has a better hit rate than 5eqh, this model could not reproduce poses of co-crystallized ligand structures with accuracy.

### 4.5. In Vitro Analysis of EGCG-G1 Antioxidant Effect

Cell culture was carried out on primary cells freshly isolated from a biopsy from an abdominoplasty of a 52-year-old woman volunteer. Normal Human Epidermal Keratinocytes (NHEKs) were seeded in a black plate with a glass bottom at 20,000 cells per well in 96-well plates with a type I collagen pre-coating in quadruplicate. The cells were incubated for 24 h in complete medium (Dermalife medium supplemented with Dermalife factors) at 37 °C with 5% CO_2_ for 24 h. After 24 h of culture, the cells were treated in the complete medium with the following conditions: resveratrol 50 µM as a positive control, EGCG 0.002% or EGCG-G1 0.1% equivalent to EGCG in molarity. Skin cells untreated and cultivated with the complete medium were used as negative control. The cells were then incubated again 24 h at 37 °C, 5% CO_2_. After 24 h, the 2′,7′-Dichlorofluorescin diacetate (DCFH-DA) probe was added to the wells at 50 µM for at least 30–40 min at 37 °C. The cells were then washed two times with PBS buffer and treated with the oxidative stress inducer TBP (tert-Butyl hydroperoxide solution) at 5 mM in PBS buffer. The untreated cells remained in PBS buffer. Finally, the emitted fluorescence was measured in darkness by excitation wavelength at 488 nm and emission wavelength 525 nm with a microplate reader (TECAN).

### 4.6. Ex Vivo Analysis of EGCG-G1 Effect on Melanogenesis

#### 4.6.1. Skin Culture and Melanin Staining

Human fresh skin explants coming from a donor aged 33 years old with phototype V were used in this study (CTI Biotech, Meyzieu, France). The skin explants were topically treated for 5 days with EGCG at 0.01% or EGCG-G1 at 0.4%, or not for the untreated condition. Treatment with kojic acid at 2% was used as the positive control. Culture medium (Givaudan, Pomacle, France) was renewed every day. After sampling, the skin explants were fixed in formalin before being dehydrated and embedded in paraffin for Fontana–Masson staining. Sections of 4 μm thick were dewaxed and then stained by the Fontana–Masson silver method for melanin. Images were collected with an Axio Observer Inverted fluorescence microscope (Zeiss, Jena, Germany) in bright field mode.

#### 4.6.2. Quantification of Melanin Content by Imagery Analysis

Photomicrographs (jpeg format) of Fontana–Masson tissue sections were opened in the GIMP- GNU Image Manipulation Program. The brown-black color signals corresponding to melanin grains on stained sections were selected, copied and pasted into a new image, and saved as a jpeg file; this jpeg file consists solely of black/brown (melanin grains) on a white background. This image was subsequently opened using the ImageJ program (v1.54f). A histogram of the image was created that separates the total number of pixels in the image into 255 color categories spanning the visible spectrum. The peak corresponding to the brown-black color (melanin) was determined by cutting and summing the appropriate counts from each channel of the melanin peak. Alternatively, the numbers corresponding to the pigment peak could be pasted into an Excel spreadsheet and summed. The pigmentation index was obtained by taking the absolute number of black pixels in a representative 20X field as determined by Image J and multiplying it by 10^−3^.

### 4.7. Gene Expression Study on EGCG-G1 on a Melanocytes/Keratinocytes Coculture Model

The cell culture was realized with primary cells isolated from biopsies of a white 7-year-old male donor. The cells were co-cultured with a ratio of 10 normal human epidermal keratinocytes (NHEK) to 1 normal human epidermal melanocyte (NHEM). The experiment was carried out under 2 conditions: NHEK-NHEM coculture untreated, and NHEK-NHEM coculture + EGCG-G1 0.01% diluted in appropriate medium. The cells were seeded in 6-well plates in the presence of an appropriate culture medium for 24 h. After seeding, a treatment in the presence of EGCG-G1 was applied for 24 h. After treatment, total RNA was extracted from cells with TRIzol™ and mRNA was reverse-transcribed into cDNA. cDNA was mixed with different primers representative of the melanogenesis pathway (PrimePCR custom plate, pigmentation, Bio-Rad). Quantification of mRNA was achieved by semi-quantitative PCR on Bio-Rad CFX96 Real Time PCR System using SsoAdvanced™ Universal SYBR Green Supermix (Bio-Rad, Marnes-la-Coquette, France). The gene expression level was calculated and normalized with reference gene (β2-Microglobulin). The values were reported to the untreated control to express an activating or inhibitory effect. 

### 4.8. Impact of EGCG-G1 on Bacillus sp. and Lactobacillus sp. In Vitro

#### 4.8.1. Analysis of EGCG-G1 Composition Modification by *Bacillus* sp.

*Bacillus* sp. (reference GAB04-01) was isolated by Givaudan on normal healthy skin. Bacterial strain was stored at −80 °C in the presence of 20% *w*/*v* glycerol. A selected suspension was thawed and an aliquot was spread on a Tryptic Soy Agar plate before incubation at 30 °C. A single colony was used to inoculate 10 mL of Tryptic Soy Broth (TSB) and incubated at 37 °C overnight under agitation. The optical density at 600 nm was measured after 10-time dilution with TSB medium (Biophotometer D30). An aliquot of the suspension was first diluted with a fresh culture medium (DSM 0.5) to obtain an optical density of 1.216. A volume of 1 mL of this suspension was then used to inoculate 40 mL of fresh culture medium (DSM 0.5). The optical density of the reaction medium just after inoculation was 0.030 ± 0.003. After 24 h of culture, 1% of EGCG-G1 was added to the medium. Addition of water was used for the negative control. The conversion of EGCG-G1 by *Bacillus* sp. was followed using HPLC-UV-MS (Ultimate 3000 System, ThermoFisher Scientific, Courtabœuf, France) with the following parameters: 1.00 mL/min, column X-Bridge C18 (Waters), injection: 10 µL; oven temperature: 40 °C; UV wavelength: 270 nm. 

#### 4.8.2. Effect of EGCG-G1 on *Lactobacillus acidophilus*

*L. acidophilus* (DSM 20079 isolated from human) was cultivated at 37 °C under anaerobia on De Man, Rogosa Sharpe (MRS) agar. After bacterial amplification in the culture medium, a bacterial suspension was prepared using the assay medium (MRS broth 50% in PBS) and adjusted to an optical density at 600 nm (OD600 nm) of 0.2. The bacterial suspension was then transferred to 96-well plates containing or not the test compounds from 0.015% to 1%. The strain cultivated with the powdered milk at 0.01% served as a positive control. OD600 nm of bacterial suspensions was then measured at approximatively 0.1. Bacterial cultures were incubated at 37 °C under agitation (approx. 280 rpm) and under anaerobic condition. OD600 nm were read in kinetic for 48 h using a microplate spectrophotometer (EPOCH 2, BioTek Instruments) to analyze the bacterial growth. All experimental conditions were performed in triplicate (*n* = 3).

The supernatant of the *L. acidophilus* culture with and without EGCG-G1 at 1% were kept and frozen at −80 °C before analysis using high-field NMR. Supernatants of the untreated and EGCG-G1 1% treated cultures were compared. For each of these 2 conditions, 3 samples were analyzed to assess the tests reproducibility (*n* = 3). For each sample, 400 µL of supernatant were taken and 100 µL of D_2_O/TSP (3-(trimethylsilyl)propionic-2,2,3,3-d4 acid sodium salt, internal reference) were added at a concentration of 1 g/L. Then, the samples were analyzed by high-field ^1^H NMR on a Bruker ASCEND 500 spectrometer operating at a proton frequency of 500 MHz using a 5 mm cryoprobe. NMR spectra were calibrated to TSP-d4 at 0 ppm using Topspin software version 4.3.0 (Bruker Biospin, Ettlingen, Germany). Quantification of metabolites was performed by utilizing the internal standard (TSP-d4) added at a known concentration. The compounds were quantified by the relative ratio of the intensities of their peak integrals and those of the internal standard.

### 4.9. Clinical Evaluation of EGCG-G1 vs. Vitamin C on Face in Asian Skin (Study 1) 

#### 4.9.1. Panel Description

A double-blinded, placebo-controlled clinical study was carried out on 38 women volunteers aged between 45 and 75 years old with Asian skin type. The volunteers were recruited with the following inclusion criteria: hyperpigmented spots on face. The volunteers gave their informed consent for participating in this study. The volunteers were divided in 2 groups, as follows: group 1, 19 volunteers with average age of 54 ± 5 years old; group 2, 19 volunteers with average age of 53 ± 8 years old. One group applied active at 0.4% or placebo in hemi face and the other applied placebo or Vitamin C at 2% in hemi face. The volunteers applied the cream twice a day, every morning and evening, for 56 days. The brightening effect was analyzed on the face before application (D0), and after 28 days (D28) and 56 days (D56) by Colorface^®^ analysis.

#### 4.9.2. INCI Formula of Face Creams

Placebo formula was as follows: WATER, CETYL ALCOHOL, GLYCERYL STEARATE, PEG-75 STEREATE, CETEH-20, STEARETH-20, ISODECYL NEOPENTANOATE, PHENOXYETHANOL, 1.2 HEXANEDIOL, CAPRYLYL GLYCOL, DIMETHICONE, FRAGRANCE. 

Active formula was as follows: WATER, CETYL ALCOHOL, GLYCERYL STEARATE, PEG-75 STEREATE, CETEH-20, STEARETH-20, ISODECYL NEOPENTANOATE, EPIGALLOCATHECIN GALLATYL GLUCOSIDE, PHENOXYETHANOL, 1.2 HEXANEDIOL, CAPRYLYL GLYCOL, DIMETHICONE, FRAGRANCE.

Formula containing vitamin C was as follows: WATER, CETYL ALCOHOL, GLYCERYL STEARATE, PEG-75 STEREATE, CETEH-20, STEARETH-20, ISODECYL NEOPENTANOATE, ASCORBIC ACID, PHENOXYETHANOL, 1.2 HEXANEDIOL, CAPRYLYL GLYCOL, DIMETHICONE, FRAGRANCE.

#### 4.9.3. Skin Color Analysis by Colorface^®^ Technology

ColorFace^®^ is a 2D acquisition system (Newtone-Qima Life Sciences, Gençay, France) for taking standardized and multimodal photos of the entire face. The device is equipped with a 24 M pixel sensor and specific UV lamp. The acquisition methods are as follows: UltraViolet, photo without filter, crossed polarized photo, parallel polarized photo, standard 45° photo, standard 60° photo. An analysis of radiance (combination of luminosity and pigment homogeneity) and pigment defects was then carried out by Spincontrol from the photos using image analysis software. The 2D photos of the face were taken before application (D0), and after 28 days (D28) and 56 days (D56) of application.

### 4.10. Clinical Evaluation of EGCG-G1 vs. EGCG on Face in African Skin (Study 2)

#### 4.10.1. Panel Description

A double-blinded, placebo-controlled clinical study was carried out on 85 women volunteers from 18 to 47 years old with African skin. The volunteers were recruited with the following inclusion criteria: dull skin with hyperpigmentation heterogeneity on skin face. The volunteers gave their informed consent for participating in this study. The volunteers were divided in 3 groups as follows: group 1, 27 volunteers aged 29.3 ± 3.8 years old who applied active at 0.4%; group 2, 34 volunteers aged 28.7 ± 4.8 years old who applied native EGCG at 0.01%; group 3, 24 volunteers aged 29.5 ± 5.5 years old who applied placebo formula. The volunteers applied cream twice a day, every morning and evening, for 56 days. The brightening effect of the formulas was analyzed on the face with overall and localized measurements before application (D0), after 14 days (D14), after 28 days (D28) and after 56 days (D56) of application.

#### 4.10.2. INCI Formula of Face Creams

Placebo formula was as follows: WATER, CETOSTEARYL ALCOHOL, ARLACEL 165, PETROLEUM JELLY, MINERAL OIL, KEMABEN II, TEA, CARBOPOL 980.

Formula containing EGCG was as follows: WATER, CETOSTEARYL ALCOHOL, ARLACEL 165, PETROLEUM JELLY, MINERAL OIL, KEMABEN II, EPIGALLOCATHECIN GALLATYL, TEA, CARBOPOL 980.

Formula containing EGCG-G1: WATER, CETOSTEARYL ALCOHOL, ARLACEL 165, PETROLEUM JELLY, MINERAL OIL, KEMABEN II, EPIGALLOCATHECIN GALLATYL GLUCOSIDE, TEA, CARBOPOL 980.

#### 4.10.3. Overall and Localized Skin Color Analysis by Chromameter^®^

The results achieved were obtained by the mean of a Chromameter CR400, which measures skin color and parameter L* (lightness). At least three 3 (localized) and 15 readings (overall) were taken at each test sub-site for each time interval to serve as the specific spot pigmentation measurement component of this study. The greater the value, the lighter the skin color is. Skin color was analyzed at D0, D14, D28 and D56.

### 4.11. Clinical Evaluation of EGCG-G1 vs. EGCG on Face in Indian Skin (Study 3)

#### 4.11.1. Panel Description

A double-blinded, placebo-controlled clinical study was carried out on 38 women volunteers from 18 to 30 years old with Indian skin. The volunteers were recruited with the following inclusion criteria: melanin-rich skin. The volunteers gave their informed consent for participating in this study. The volunteers were divided in two groups as follows: group 1, 20 volunteers with an average age of 22 ± 3.8 years old who applied placebo; group 2: 18 volunteers with an average age of 22 ± 3.7 years old who applied formula containing EGCG-G1 at 0.4%.

The volunteers applied the cream product once a day for 56 days on the whole face. The brightening effect of the product was analyzed on the face with overall and localized measurements before application (D0), and after 14 days (D14), 28 days (D28) and 56 days (D56) of application.

#### 4.11.2. INCI Formula of Face Creams

Placebo formula was as follows: WATER, CETYL ALCOHOL, GLYCERYL STEARATE, PEG-75 STEREATE, CETEH-20, STEARETH-20, ISODECYL NEOPENTANOATE, PHENOXYETHANOL, DIMETHICONE, FRAGRANCE.

Active formula was as follows: WATER, CETYL ALCOHOL, GLYCERYL STEARATE, PEG-75 STEREATE, CETEH-20, STEARETH-20, ISODECYL NEOPENTANOATE, PHENOXYETHANOL, EPIGALLOCATHECIN GALLATYL GLUCOSIDE, DIMETHICONE, FRAGRANCE.

#### 4.11.3. Overall Skin Color Analysis by L* and ITA Parameters by Using VISIOFACE^®^

This technique consists of obtaining high-resolution photographs of ¾ of the face in completely reproducible lighting conditions in cross-polarized light. The acquisitions are carried out with a high-resolution camera. The lens used is a Nikkon 60 mm equipped with a filter. Lighting is provided by one flashlight. The flash head is fitted with filter slot to hold polarizing gel (HN32, Sarelec, France).

The filter on the lens is oriented to a 90° angle in comparison with the filter on the flash. The polarized light, emitted by the flash and reflected by the face at the moment the photo is taken, is “cut” by the filter. These reflections do not appear on the photograph when it is obtained, enabling a better visualization of the changes in skin color on the various studied areas. Using dedicated software developed by Spincontrol, the L*, a* and b* parameters are determined from the RGB values of the digital images on regions of interest defined on the face. These parameters were used to evaluate the in vivo effects of the tested product on the color of the skin. This method enables us to follow the relative evolution of the following parameters on each area: L*: brightness; a*: chromaticity coordinate representing the balance between red and green; b*: chromaticity coordinate representing the balance between yellow and blue; and ITA°: individual typological angle (=ATAN[(L* − 50)/b*] × (180/π)).

#### 4.11.4. Localized Skin Color Analysis by Chromameter^®^

Tristimulus colorimeters are made of a control unit and a measurement head line. The measurement head line takes the measurements from a zone of 8 mm in diameter and uses diffuse lighting as well as an angle of 0° (including specular component). The headline measurement has a light with a pulsed xenon arch lamp. A double-beam system measures the incident light and the reflected light by means of six photocells. The used material is a Chromameter CR-400 (Minolta, Osaka, Japan).

Brown spots’ analysis was realized by three measurements on 3 different areas on one cheek. One measurement per area was realized to determine uneven skin tone. The site of the instrumental measurements and their location at the different points of the kinetics was strictly the most reproducible. The location was determined by a cutaneous marking on the instrumental measurement sites at day 0 (before application). In order to place this marking accurately, a mapping of the skin’s surface, for the measurement site and for each subject, was made on a transparent film. The absolute measurements were expressed in the form of L*, a* and b* and ITA°. L* represents the relative brightness from total black (L* = 0) to total white (L* = 100). ΔE* represents the relative difference in color between two given areas, and it is calculated as follows: ΔE* = [(L*si − L* sj)2 + (a*si − a*sj)2 + (b*si − b*sj)2 ]1/2, where si and sj means two different sites defined on the face.

For each absolute measurement, the mean of the three measurements was calculated for each site. The color differences between the different investigated sites were calculated and plotted. A significant increase in L* and ITA° parameter corresponds to an improvement in skin brightening. A significant decrease in the ∆E parameter corresponds to an improvement in the skin tone evenness (skin homogeneity). This measurement was carried out before application (D0), and after 14 days (D14), 28 days (D28) and 56 days (D56) of application.

### 4.12. Clinical Evaluation of EGCG-G1 on Hands and Cheeks in Caucasian Skin (Study 4)

#### 4.12.1. Panel Description

A double-blinded, placebo-controlled clinical study was carried out on 30 women volunteers aged between 45 and 75 years old with white skin type. The volunteers were recruited with the following inclusion criteria: hyperpigmented spots on hands; brown spots and sagging on face. The volunteers gave their informed consent for participating in this study. The volunteers were divided in 2 groups as follows: group 1, 15 volunteers with an average age of 60 ± 5.7 years who applied the active (EGCG-G1) at 0.4%; group 2, 15 volunteers with an average age of 64 ± 7.0 years who applied placebo formula as the control. The volunteers applied cream twice a day, every morning and evening, for 56 days on whole face and on hands. The reduction in melanin content on hyperpigmented area was analyzed on the hand before application (D0) and after 56 days of application (D56) by autofluorescence of melanin using Raman. Samples of microbiota were collected from the cheek of 30 volunteers, by a non-invasive swabbing method, using sterile swabs moistened with a sterile solution of 0.15 M NaCl. The swabs were transferred at −20 °C and kept frozen until DNA extraction. Sampling was carried out before treatment (D0), and after 28 days (D28) and 56 days (D56) of treatment, using a standardized procedure.

#### 4.12.2. INCI Formula of Hand and Face Creams

Placebo formula was as follows: WATER, CETYL ALCOHOL, GLYCERYL STEARATE, PEG-75 STEREATE, CETEH-20, STEARETH-20, ISODECYL NEOPENTANOATE, PHENOXYETHANOL, 1.2 HEXANEDIOL, CAPRYLYL GLYCOL, DIMETHICONE, FRAGRANCE.

Active formula was as follows: WATER, CETYL ALCOHOL, GLYCERYL STEARATE, PEG-75 STEREATE, CETEH-20, STEARETH-20, ISODECYL NEOPENTANOATE, EPIGALLOCATHECIN GALLATYL GLUCOSIDE, PHENOXYETHANOL, 1.2 HEXANEDIOL, CAPRYLYL GLYCOL, DIMETHICONE, FRAGRANCE.

#### 4.12.3. Hand Skin Color Analysis by Measuring Melanin Autofluorescence by Raman Spectroscopy

The set-up included a confocal Raman probe coupled to a dispersive Raman spectrograph. The excitation laser beam was sent to the remote probe via a 5 µm core-diameter fiber and the Raman signal was conveyed to the spectrograph via 100 µm core-diameter fiber. The probe was equipped with a 100× long working distance objective operating in air. A piezo-electric device allowed us to collect Z Raman profiles by assuring axial measurements from the surface down to a defined depth in the skin. A color video camera integrated in the probe allowed us to visualize the skin surface. This camera is also useful to control the focalization of the laser on the skin. The spectrograph was equipped with a CCD (Coupled Charge Detector) of 1024 × 256 elements cooled by Peltier effect, and an 830 grooves/mm grating, which allows us to cover a large spectral range from 400 to 3620 cm^−1^ in a single shot acquisition with a spectral resolution of about 6 cm^−1^. The excitation source was a 660 nm laser diode and the power at the sample was fixed at 30 mW in accordance with protection standards for radiation. For data acquisition, the device was controlled by the Labspec 5 software. 

The pre-processing of spectral data was performed using Matlab 7.2 (The MathWorks Inc., Natick, MA, USA). Aberrant profiles (bad S/N (signal/noise) ratio, fluorescence saturation, incomplete profile with a large zero offset, etc.) were excluded from the data base. Each non-aberrant profile was submitted to background corrections, which allowed us to clean up the Raman signal of the skin. These background corrections included smoothing spectra with a 9 mm Savitzky–Golay filter, spike correction and the normalization on the intensity of the entire wavenumber range, with a vectorial function.

A baseline correction was not applied in order to preserve the skin background fluorescence, which allowed for the extraction of the skin fluorescence markers associated with the degree of the skin lightening. 

After determination of the SC surface spectra on each (Z) profile in the spectral database, the next step was to determine the spectroscopic marker specific to the skin background fluorescence. For this, the comparison of Raman spectra measured at D0 on both areas, unstained area (Tém) and brown spot (TB), was carried out. To estimate the spectral difference between the Raman spectra of the unstained area (Tém) and the brown spot (TB) at D0, the spectra were submitted to a statistical analysis to determine if there were spectral ranges with significant differences between unstained and spotted skin. Two measurements per area (brown spots and skin tone) were made before application (D0) and after 56 days of application (D56).

#### 4.12.4. DNA Extraction, Microbial DNA Sequencing and Data Analysis 

DNA extraction was performed, for 102 samples, using the DNeasy PowerLyzer^®^ PowerSoil^®^ DNA Isolation Kit with Qiacube device (Qiagen, Hilden, Germany), with the following modifications. The tip of each swab was detached with a sterile surgical blade and transferred into a 1.5 mL tube containing 750 μL of Bead Solution. The sampled biomass was suspended by stirring and pipetting, and then transferred to a bead beating tube. The remaining steps were performed according to the manufacturer’s instructions. DNA concentration was determined using the QuBit dsDNA HS fluorometric quantitation kit (Invitrogen, ThermoFisher Scientific, Courtaboeuf, France) according to the manufacturer’s instructions.

Sequencing was performed with the MiSeq device (Illumina, Inc., San Diego, CA, USA) through a 500 cycle paired-end run, targeting the V3V4 16S variable regions using the following primers: 16S-Mi341F forward primer 5′- CCTACGGGNGGCWGCAG-3′ and 16S-Mi805R reverse primer 5′-GACTACHVGGGTATCTAATCC-3′, producing about 460 bp amplicons. PCR1s were performed as follows: 8 μL of template DNA (0.2 ng) was mixed with 5 μL of each reverse and forward primers (1 μM), 5 μL of KAPA HiFi Fidelity Buffer (5×), 0.8 μL of KAPA dNTP Mix (10 mM each), 0.7 μL of RT-PCR-grade water (Ambion), and 0.6 μL of KAPA HiFi hotstart Taq (1 U/μL), for a total volume of 25 μL. Each amplification was duplicated, and the duplicates were pooled after amplification. PCR1 cycles consisted of 95 °C for 3 min and then 32 cycles of 95 °C for 30 s, 59 °C for 30 s, and 72 °C for 30 s, followed by a final extension at 72 °C for 3 min, with an eppendorf Nexus thermocycler (Eppendorf, Hambourg, Germany). Negative and positive controls were included in all steps to check for contamination. All duplicate pools were controlled by gel electrophoresis, and amplicons were quantified using fluorometry. Libraries ready for analysis were then produced following the Illumina guidelines for 16S metagenomics libraries’ preparation. Briefly, the PCR1 amplicons were purified and controlled using an Agilent 4200 TapeStation system (Agilent Technologies, Santa Clara, CA, USA). To enable the simultaneous analysis of multiple samples (multiplexing), Nextera^®^ XT indexes (Illumina) were added during PCR2 using between 15 and 30 ng of PCR1 amplicons. PCR2 cycles consisted of 94 °C for 1 min and then 12 cycles of 94 °C for 60 s, 65 °C for 60 s, and 72 °C for 60 s, followed by a final extension at 72 °C for 10 min (eppendorf Nexus thermocycler). Indexed libraries were purified, quantified and controlled using an Agilent 4200 TapeStation system. Validated indexed libraries were pooled in order to obtain an equimolar mixture. The run (500 cycles) was achieved on MiSeq sequencer (Illumina) using the MiSeq Reagent Kit v3 600 cycles (Illumina). The sequencing run produced an output of 15.3 million of paired-end reads of 250 bases, i.e., up to 3.8 Gigabases. The libraries and the MiSeq run were performed at the GeT-PlaGe platform (INRA, Auzeville, France).

After MiSeq run, raw data sequences were demultiplexed and quality-checked to remove all the reads with ambiguous bases. Indexes and primers sequences were removed with cutadapt (v3.3; http://cutadapt.readthedocs.io/en/stable/index.html accessed on 8 March 2021) and reads with a fastq score lower than 28 were trimmed. The forward and reverse sequences were paired using bbmerge (https://jgi.doe.gov/data-and-tools/bbtools/ accessed on 15 January 2022). Samples with less than 5000 paired sequences were discarded. The remaining paired sequences were then treated using an in-house pipeline that uses vsearch [61] to remove chimeras and amplicons with PCR errors. Sequences were then split into Operational Taxonomic Units (OTUs, a cluster of similar sequence variants of the 16S rRNA marker gene sequence) at a 1% dissimilarity level using swarm (v2.6—[62]). Unique amplicons were mapped to the SILVA SSU Ref NR 99 (non-redundant) database (release 138.1; https://www.arb-silva.de/ accessed on 7 September 2020) for taxonomic assignation using the RDP classifier [63]. Data normalization and analyses were carried out using R statistical computing environment (v4.1.2; https://www.r-project.org/ accessed on 5 November 2021—R core team (2014) using Bioconductor package (mainly Phyloseq, DESeq2 and Vegan libraries; v3.13 http://www.bioconductor.org/ accessed on 18 October 2021).

Data were then compared using Wilcoxon’s test for paired samples (Wilcoxon, 1945). Due to multiple testing, *p*-value were adjusted using false discovery rate (FDR) correction [64].

### 4.13. Statistical Analysis

For in vitro and ex vivo experiments, a Shapiro–Wilk normality test was performed to evaluate whether the data follow the Gaussian Law. If the results did not follow the Gaussian Law, a non-parametric statistical analysis was performed by Kruskal–Wallis ANOVA followed by Mann–Whitney U test. Otherwise, Student’s *t* test was used.

For all in vivo studies, a Shapiro–Wilk test was used to verify whether the raw data followed the Gaussian Law. In case of normally distributed data, the mean values were compared using either an unpaired or paired Student’s *t* test. In case of non-normally distributed data, a Wilcoxon (paired) or Kruskal–Wallis test followed by a Mann–Whitney U (unpaired) test were used for paired data or unpaired data, respectively.

Regardless of the test, it was considered a significant result; *p* < 0.1 with #, *p* < 0.05 with *, *p* < 0.01 with **, *p* < 0.001 with ***.

## 5. Conclusions

Enzymatic glucosylation of EGCG promoted its skin penetration, and possibly its bioavailability, due to a potential interaction with GLUT1 transporter. The brightening properties of EGCG-G1 were demonstrated on in vivo studies conducted in four countries, on hyperpigmented spots and on full face skin tone. Glucosylation allowed us to maintain the photoprotective antioxidant activity of EGCG. Antioxidant properties of EGCG-G1 contributed to its anti-melanogenic activity, which was found to depend on the down-regulation of genes involved in melanin synthesis and indirectly by stimulation of Lactobacillus sp., which could produce a brightening microbial metabolite under EGCG-G1 stimulation. This study provides an exhaustive demonstration of EGCG-G1’s effects on hyperpigmentation in vivo, and provides new data on the biological mechanisms underlying EGCG-G1’s mode of action due to its holistic approach.

## 6. Patents

The patent WO 2024/100140 resulted from the work reported in this manuscript.

## Figures and Tables

**Table 1:** Gene expression study on expression of genes involved in the melanogenesis by a co-culture of keratinocytes and melanocytes following treatment with EGCG-G1 and compared to untreated co-culture. KIT: Tyrosine kinase receptor, EDNRB: Endothelin receptor type B, MC1R: Melanocortin 1 receptor, SOX10: Sex-determining region Y-box 10, MITF: Microphthalmia-associated transcription factor, TYR: Tyrosinase, TYRP1: Tyrosinase-related protein1, AP3B1: Adaptor-related protein complex 3 subunit beta 1, GPR143: G protein-coupled Receptor 143, PMEL: Melanocyte specific protein, DTNBP1: Dystrobrevin Binding Protein 1, LYST: Lysosomal Trafficking regulator, F2RL1: F2R like trypsin receptor 1. * *p* < 0.05, ** *p* < 0.01, *** *p* < 0.001.

Step of Melanogenesis Regulation	Gene	Fold Change vs. Untreated	% vs. Untreated and *p*-Value
Regulation of signaling pathways	*KIT*	0.52	−93% ***
	*EDNRB*	0.6	−68% ***
	*MC1R*	0.51	−96% ***
	*SOX10*	0.62	−60% ***
	*MITF*	0.48	−108% ***
Melanin synthesis	*TYR*	0.63	−58% ***
	*TYRP1*	0.51	−95% **
Melanosome biogenesis	*AP3B1*	0.63	−59% *
	*GPR143*	0.21	−365% ***
	*PMEL*	0.32	−212% ***
	*DTNBP1*	0.63	−59% *
	*LYST*	0.67	−48% ***
Melanin uptake	*F2RL1*	0.67	−49% ***

**Table 2:** Overview of parameters describing clinical trials that evaluated the impact of EGCG-G1 on skin pigmentation and microbiota. Main results are also briefly summarized. All studies are double-blinded and placebo-controlled and were conducted for 56 days. Vehicles had the same formulation except in study 3. Study nb: Study identification number; Skin type: ethnicity; Nb vol. (Nb/group): Number of volunteers (Number of volunteers per group); Skin area for appl.: skin area where cream was applied and measures performed; Age range: age range of the panelists; Inclusion criteria: inclusion criteria of study concerning skin pigmentation; Products tested by groups: indicate actives tested vs. placebo in cream; Frequency of appl.: number of application of cream per day; Tools: apparatus or method used to evaluate skin; 🡕: indicates an increase in parameter; 🡖: indicates a decrease in parameter; L*: lightness; ITA: individual typology angle; ΔE: color variation between two areas.

Study Nb.	Skin Type	Nb Vol. (Nb/Group)	Skin Area for Appl.	Age Range (Years)	Inclusion Criteria	Products Tested by Groups	Frequency of Appl.	Tools	Effect Of EGCG-G1 0.4%
**1**	Asian	38 (19,19)	Face (hemiface)	45–75	Hyperpigmented spots	EGCG-G1 0.4%/placebo Vitamin C 2%/placebo	2	Colorface	🡕 radiance 🡕 skin homogeneity 🡖 surface pigment defect Efficacy > Vitamin C 2%
**2**	African	85 (27,34,24)	Face (whole face)	18–47	Dull skin, hyperpigmentation, skin heterogeneity	EGCG-G1 0.4% EGCG 0.01% Placebo	2	Chromameter	🡕 L* on brown spots 🡕 L*whole face 🡖 uneven skin tone Efficacy >> EGCG
**3**	Indian	38 (20,18)	Face (whole face)	18–30	Melanin-rich skin	EGCG-G1 0.4% Placebo	1	Visioface Chromameter	Chromameter: 🡕 L* 🡕 ITA 🡖 ΔE Visioface: 🡕 L* 🡕 ITA
**4**	Caucasian	30 (15,15)	Hands	45–75	Hyperpigmented spots	EGCG-G1 0.4% Placebo	2	Raman probe	🡖 melanin in brown spots 🡖 skin heterogeneity
			Face		Brown spots, sagging			Microbiota sampling, 16S rRNA sequencing	🡕 *Lactobacillus* population

## Data Availability

The original contributions presented in the study are included in the article, further inquiries can be directed to the corresponding author.

## Supplementary Materials

The following supporting information can be downloaded at: https://www.mdpi.com/article/10.3390/molecules29225391/s1, Figure S1: Chemical shifts 1H and 13C in (-)-epigallocatechin-3-gallate-4′-O-α-D-glucoside; Figure S2: Glucose Binding Site of the GLUT1 transporter; Figure S3: Illustrative pictures of one panelist participating in the clinical study on Asian mature skin; Figure S4: Illustrative pictures of one panelist participating in the clinical study on African skin; Figure S5: Illustrative pictures of one panelist participating in the clinical study on Indian skin; Figure S6: Illustrative pictures of one panelist participating in the clinical study on white hand skin.
