# Supplementary material for: Epigallocatechin Gallate Enzymatic Alpha Glucosylation Potentiates Its Skin-Lightening Activity—Involvement of Skin Microbiota
Source: Molecules. 2024 Nov 15;29(22):5391. doi: 10.3390/molecules29225391 (PMC11597265; doi:10.3390/molecules29225391)
Supplement: Supplementary file 1 [file molecules-29-05391-s001.zip › Supplementary Material Figure S1.pdf]

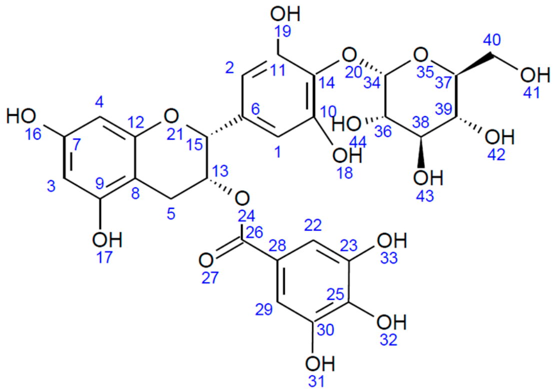

Atom number	^{13}C (ppm)	^{1}H (ppm)
1	105.8	6.48
2	105.8	6.48
3	94.7	5.85
4	96.0	5.94
5	26.1	2.67/2.94
6	135.5	-
7	157.0	-
8	97.8	-
9	155.9	-
10	150.6	-
11	150.6	-
12	156.9	-
13	68.2	5.39
14	133.7	-
15	76.7	5.02
22	109.0	6.81
29	109.0	6.81
23	145.9	-
30	145.9	-
25	139.0	-
26	165.4	-
28	119.5	-
34	104.3	4.83
36	72.2	3.38
37	74.3	3.98
38	73.6	3.59
39	69.8	3.23
40	60.5	3.55/3.61

Figure S1. Chemical shifts ^{1}H and ^{13}C of (-)-epigallocatechin-3-gallate-4′-O-α-D-glucoside (CAS 1618020305, MW 620.54 g/mol) in DMSO-*d6* determined by 13C NMR, HSQC, HMBC and COSY experiments.
