# Supplementary material for: Epigallocatechin Gallate Enzymatic Alpha Glucosylation Potentiates Its Skin-Lightening Activity—Involvement of Skin Microbiota
Source: Molecules. 2024 Nov 15;29(22):5391. doi: 10.3390/molecules29225391 (PMC11597265; doi:10.3390/molecules29225391)
Supplement: Supplementary file 1 [file molecules-29-05391-s001.zip › Supplementary Material Figure S2.pdf]

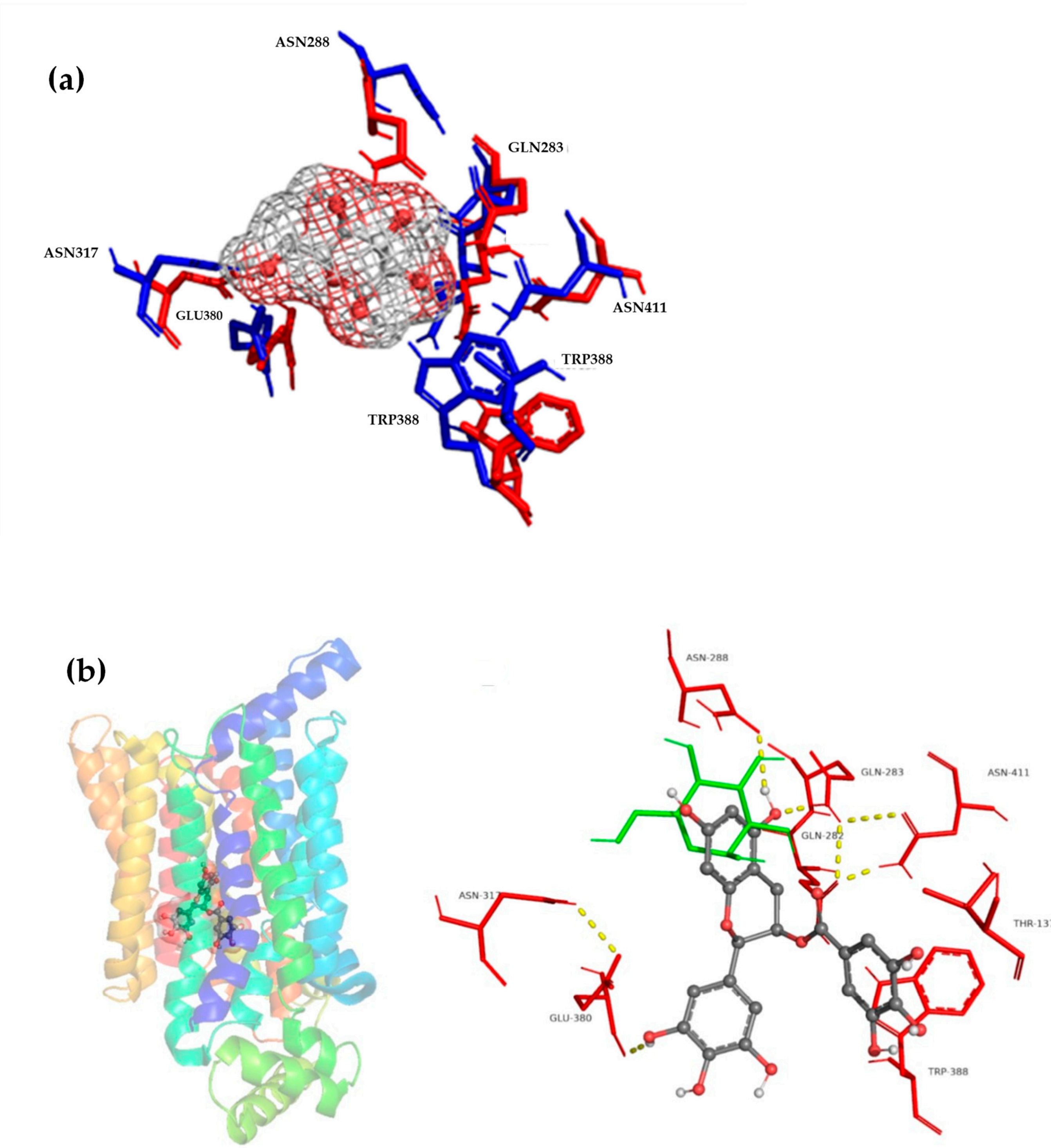
(a)
ASN288
GLN283
ASN317
GLU380
ASN411
TRP388
TRP388
(b)
ASN-288
GLN-283
ASN-411
GLN-282
ASN-317
THR-137
GLU-380
TRP-388

(c)

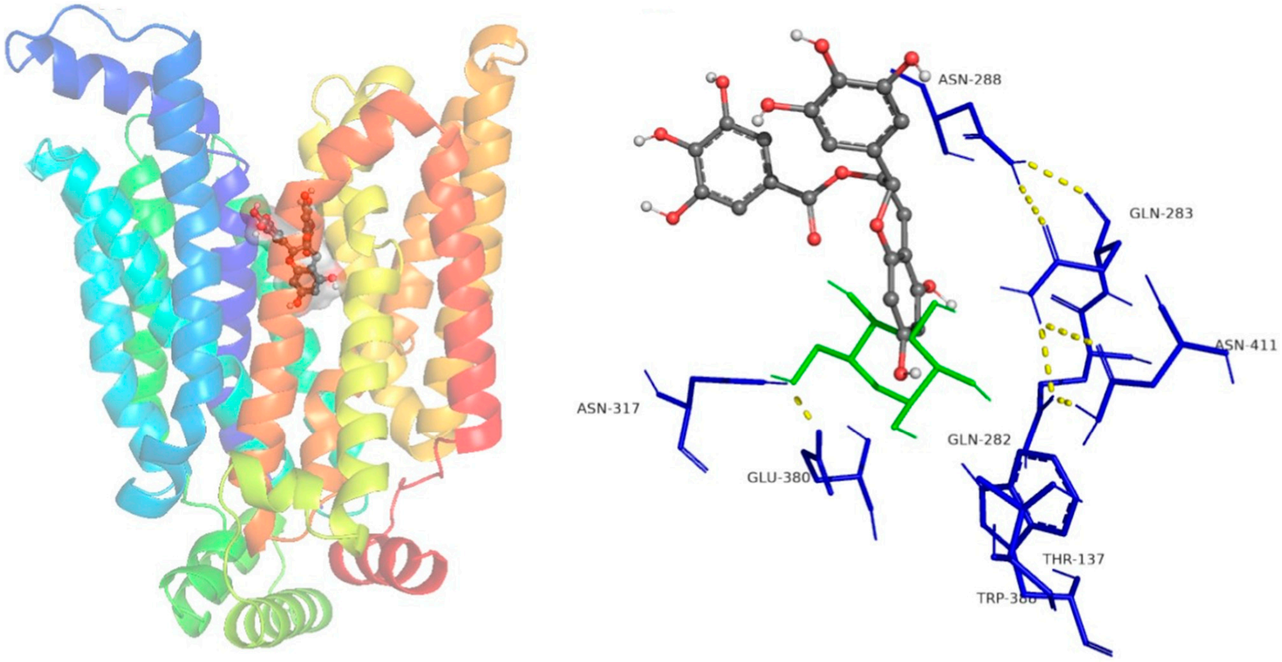
ASN-288
GLN-283
ASN-411
ASN-317
GLN-282
GLU-380
THR-137

(d)

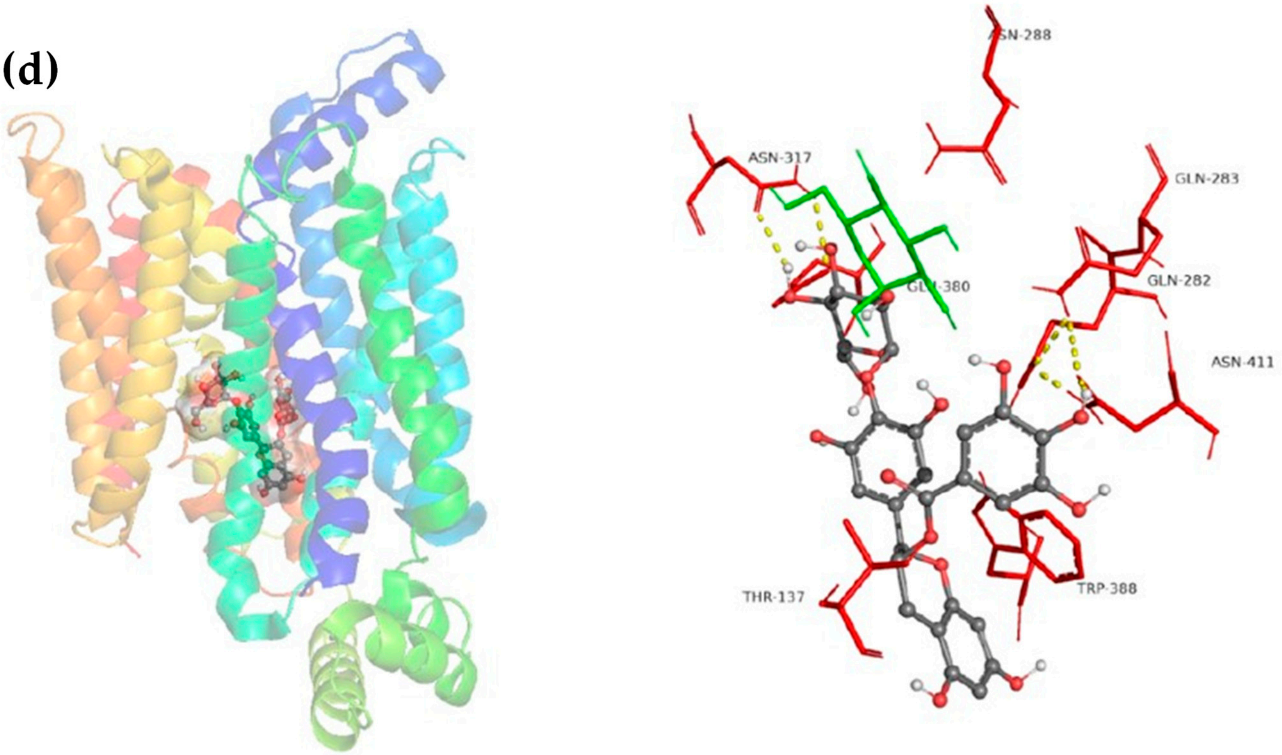
ASN-288
ASN-317
GLN-283
GLN-282
ASN-411
THR-137
TRP-388

(e)

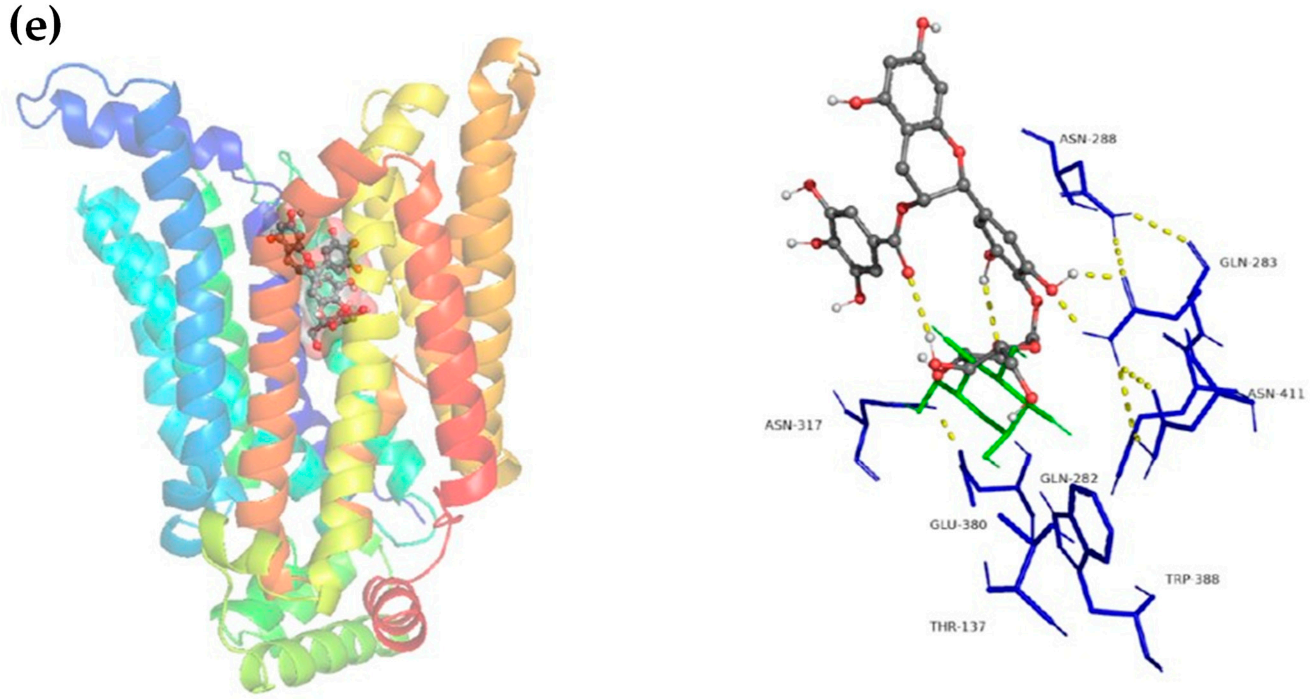

Figure S2. Glucose Binding Site of the GLUT1 transporter (A). Residues from the glucose binding site are shown in blue for the modeled outward conformation, based on the 4zwc PDB structure, and in red for the inward conformation, from the 5eqh PDB structure. The glucose, colored in white (carbon atoms) and red (oxygen atoms) is represented with sticks and balls and positioned according to the PDB structure 4zwc. The volume occupied by the glucose is shown by a mesh representation. Docking of EGCG and GLUT1 in the inward (B) and outward (C) conformation of the transporter. Docking of EGCG and GLUT1 in the inward (D) and outward (E) conformation of the transporter

Docking experiment on EGCG and EGCG-G1 with GLUT1 revealed both ligands bound the same cavity than the glucose. For the inward and outward conformations of the transporter, EGCG presented the bicycle substructure placed at the glucose position according to crystal structure, while EGCG-G1 presented the glucose moiety at this position.

In the inward conformation, EGCG interacted by H-bond with residues GLN283, ASN288 and GLU380 while EGCG-G1 interacted with residues ASN317 and ASN411. All two compounds were positioned to make π-π interaction with TRP388.

Regarding the outward conformation, EGCG interacted with the residues ASN34, GLN172 with H bound and was positioned to interact with residue PHE291 by π-π interaction. EGCG-G1 interacted at the binding site with residue GLN283 with H-bonds and outside the binding site with residues ASN34 and GLN172 with H-bonds and also could have made π-π interaction with residue PHE291.
