# Supplementary material for: Epigallocatechin Gallate Enzymatic Alpha Glucosylation Potentiates Its Skin-Lightening Activity—Involvement of Skin Microbiota
Source: Molecules. 2024 Nov 15;29(22):5391. doi: 10.3390/molecules29225391 (PMC11597265; doi:10.3390/molecules29225391)
Supplement: Supplementary file 1 [file molecules-29-05391-s001.zip › Supplementary Material Figure S4.pdf]

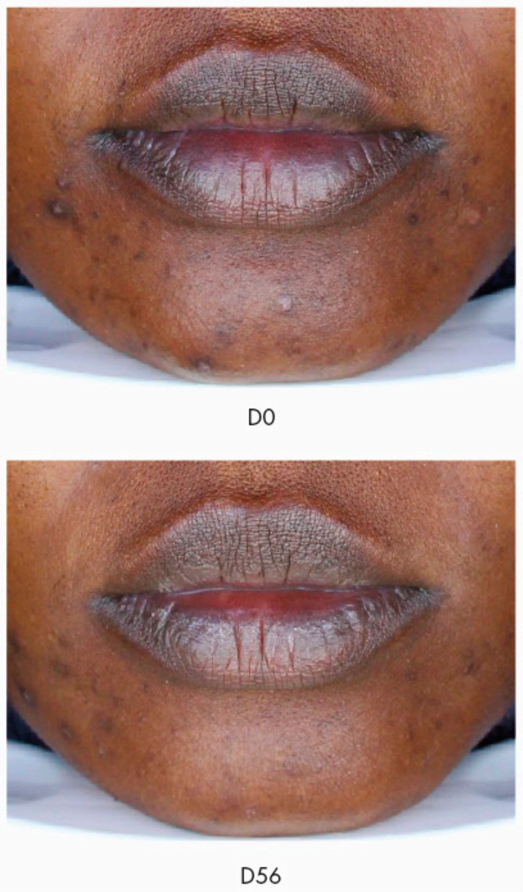

Figure S4. Illustrative pictures of one panelist participating in the clinical study on African skin and presenting hyperpigmentation disorders. Panelist applied active formula containing EGCG-G1. Pictures focus on chin zone. Upper picture presents skin aspects on day 0, before first application, and lower photography presents the same facial area after 56 days of treatment.
