# Supplementary material for: Epigallocatechin Gallate Enzymatic Alpha Glucosylation Potentiates Its Skin-Lightening Activity—Involvement of Skin Microbiota
Source: Molecules. 2024 Nov 15;29(22):5391. doi: 10.3390/molecules29225391 (PMC11597265; doi:10.3390/molecules29225391)
Supplement: Supplementary file 1 [file molecules-29-05391-s001.zip › Supplementary Material Figure S6.pdf]

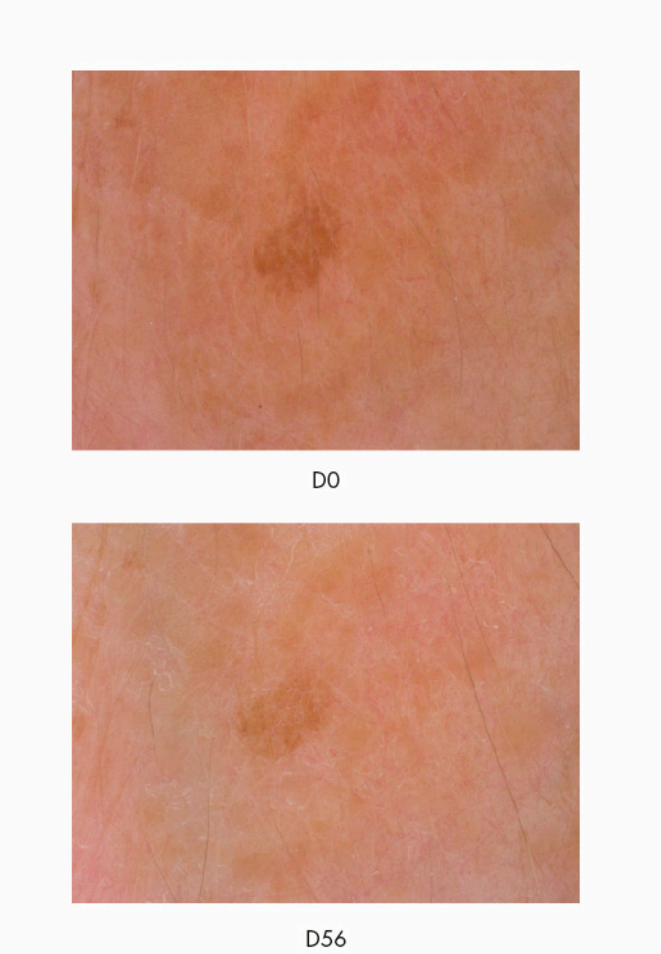

Figure S6. Illustrative pictures of one panelist participating in the clinical study on Caucasian hands skin presenting solar lentigo. Panelist applied active formula containing EGCG-G1. Pictures focus on one brown spot localized on hand. Upper picture presents skin aspect on day 0, before first application, and lower photography presents the same facial area after 56 days of treatment.
